# Evidence of Activity-Specific, Radial Organization of Mitotic Chromosomes in *Drosophila*

**DOI:** 10.1371/journal.pbio.1000574

**Published:** 2011-01-11

**Authors:** Yuri G. Strukov, Tûba H. Sural, Mitzi I. Kuroda, John W. Sedat

**Affiliations:** 1 Department of Biochemistry and Biophysics, University of California San Francisco, San Francisco, California, United States of America; 2 Division of Genetics, Department of Medicine, Brigham & Women's Hospital, Boston, Massachusetts, United States of America; 3 Department of Genetics, Harvard Medical School, Boston, Massachusetts, United States of America; National Cancer Institute, United States of America

## Abstract

The organization and the mechanisms of condensation of mitotic chromosomes remain unsolved despite many decades of efforts. The lack of resolution, tight compaction, and the absence of function-specific chromatin labels have been the key technical obstacles. The correlation between DNA sequence composition and its contribution to the chromosome-scale structure has been suggested before; it is unclear though if all DNA sequences equally participate in intra- or inter-chromatin or DNA-protein interactions that lead to formation of mitotic chromosomes and if their mitotic positions are reproduced radially. Using high-resolution fluorescence microscopy of live or minimally perturbed, fixed chromosomes in Drosophila embryonic cultures or tissues expressing MSL3-GFP fusion protein, we studied positioning of specific MSL3-binding sites. Actively transcribed, dosage compensated *Drosophila* genes are distributed along the euchromatic arm of the male X chromosome. Several novel features of mitotic chromosomes have been observed. MSL3-GFP is always found at the periphery of mitotic chromosomes, suggesting that active, dosage compensated genes are also found at the periphery of mitotic chromosomes. Furthermore, radial distribution of chromatin loci on mitotic chromosomes was found to be correlated with their functional activity as judged by core histone modifications. Histone modifications specific to active chromatin were found peripheral with respect to silent chromatin. MSL3-GFP-labeled chromatin loci become peripheral starting in late prophase. In early prophase, dosage compensated chromatin regions traverse the entire width of chromosomes. These findings suggest large-scale internal rearrangements within chromosomes during the prophase condensation step, arguing against consecutive coiling models. Our results suggest that the organization of mitotic chromosomes is reproducible not only longitudinally, as demonstrated by chromosome-specific banding patterns, but also radially. Specific MSL3-binding sites, the majority of which have been demonstrated earlier to be dosage compensated DNA sequences, located on the X chromosomes, and actively transcribed in interphase, are positioned at the periphery of mitotic chromosomes. This potentially describes a connection between the DNA/protein content of chromatin loci and their contribution to mitotic chromosome structure. Live high-resolution observations of consecutive condensation states in MSL3-GFP expressing cells could provide additional details regarding the condensation mechanisms.

## Abstract 2

A fluorescently labeled protein specifically binding to genes was reproducibly found at the periphery of condensed mitotic fruit fly chromosomes, illustrating preservation of a radial structure between consecutive divisions.

## Author Summary

Mitotic chromosomes of eukaryotes are relatively large rod-like cellular organelles, about 1 µm in diameter and 10 µm long, of well-studied composition but unknown structure. The question of whether all DNA sequences equally contribute to the interactions leading to the formation of mitotic chromosomes has never been asked. To find an answer, we determined whether the radial positions of specific chromatin loci within mitotic chromosomes were reproduced at every cell cycle or were purely random. Based on fluorescence microscopy images of live or fixed chromosomes in cells from *Drosophila* embryos or *Drosophila* larval tissues expressing the MSL3-GFP fusion protein from a transgene, we report that the large-scale organization of mitotic chromosomes is reproduced not only longitudinally, as in the well-known chromosome banding phenomenon, but also radially. Actively transcribed, dosage-compensated genes of the *Drosophila* male X chromosome were always found at the periphery of mitotic chromosomes, starting from late prophase. Histone modifications specific to active chromatin were found to be more peripheral compared to silent chromatin that tended to be more central in the condensed chromosome. These findings are both exciting and significant for the field of cell and chromatin biology because they may help reconcile the old controversy between the existing models of chromosome structure that posit either radial loops of chromatin or consecutive coiling. In addition, we offer new insights into the mechanisms of mitotic condensation and suggest a link between structural and functional roles of different chromatin domains.

## Introduction

Over the past decades mitotic chromosomes have been shown to have a high degree of organization. However, the exact configuration of the DNA molecule and its reproducibility within a chromosome are unknown. Consolidation of the results from diverse experimental approaches has not yet led to a thorough understanding of chromosome structure. Structural features of chromosomes are beyond the resolution of light microscopy, and tight compaction and lack of contrast in electron microscopy are among the main technical obstacles [1],[2]. Even though the correlation between DNA sequence composition and its contribution to the chromosome-scale structure has been suggested before [3],[4], it is unclear if any DNA sequence is equally able to participate in intra- or inter-chromatin or DNA-protein interactions, leading to formation of mitotic chromosomes. Alternatively, some regions may be suited for this purpose more than others. Distinct models of mitotic chromosomes concentrate on different aspects of their structure [5]. Complete or partial extraction of chromosomes known to modify the native chromosome morphology has lead to the “radial-loop” model [6],[7]. The original “radial-loop” model, with its later modifications based on biochemical and cytological experiments on fully condensed mitotic chromosomes [8], postulated the existence of specialized DNA sequences anchoring chromatin loops to non-histone proteins at the cores of chromosomes approximately every 100 kbp and indispensable for a variety of other biological functions besides mitotic condensation. Models of this class do not specify the organization of the “30 nm fiber” between the anchoring points. Alternative, “hierarchical-coiling” models, based on observations of bulk chromatin at different stages of mitotic condensation with light or electron microscopy, in part due to insufficient resolution, conceptually overlook the possibility of correlation between the DNA sequence/protein composition of a specific chromatin region and its contribution to chromosome structure [9]. These models concentrate on “large-scale” structural features ranging in size from several tens to several hundred nm and therefore detectable with microscopy. Additional models with features borrowed from both “radial-loop” and “hierarchical coiling” models also have been proposed [10],[11]. Despite identification of a number of proteins necessary for successful condensation and segregation of chromosomes in mitosis, key features of the structure, among which are banding patterns, reproducible chromosome geometry, and localization of topoisomerase II or condensin complexes within chromosomes, await their consolidation and explanation by a model. The question of whether all DNA sequences equally participate in the formation of chromosomes or whether the structural role is entrusted to a narrower class of specialized sequences remains unanswered.

Here we probed the connection between the function of chromatin loci in terms of transcriptional activity and their position on mitotic chromosomes. Dosage compensated genes on the X chromosome in fruit flies provide a functionally distinct subset of genes with a possibility of labeling for fluorescence microscopy. As demonstrated by both cytological and chromosome-wide mapping studies, the euchromatic arm of the X chromosome is specifically bound by MSL complex throughout the cell cycle, including mitosis [12]–[15], providing a convenient label for *Drosophila melanogaster* chromatin in its native state in live cells. As a source of mitotic chromosomes, we used diploid dividing cells from live fly tissues and freshly isolated primary cultures from cellularized embryos expressing GFP fused with MSL3, one of the components of the *Drosophila* dosage compensation complex (DCC), also called MSL complex (Male Specific Lethal) [16]. In live embryonic cultures or live 3^rd^ instar larval tissues, specific MSL3-binding sites were detected through localization of MSL3-GFP, the feature characteristic of active genes on the male X chromosome.

We further explored the potential relationship between transcriptional activity and location of sequences within mitotic chromosomes by immunostaining for specific post-translationally modified histones [17],[18]. In a variety of organisms, both transcriptionally silent chromatin, characterized by relatively condensed DNA, and more decondensed transcriptionally active chromatin are marked by specific histone modifications [19]. Various histone marks may continuously stretch over regions of tens of kbp on the scale of gene clusters [20],[21] and remain stable over several cell cycles [22]–[24]. In *Drosophila*, methylation of histone H3 at lysine 4 is associated with actively transcribed sequences and found in interbands of polytene chromosomes. Monomethylation of lysine 27 in H3 (H3K27me1) is found at pericentric heterochromatin and in most euchromatic bands in polytene chromosomes [25]; monomethylated lysine 20 at H4 (H4K20me1) is known to associate with chromocenter heterochromatin and a high number of euchromatic bands [19].

Combining novel fluorescence microscopy techniques with improved spatial and temporal resolution [26] and labeling of specific chromatin loci on the genome scale, we were able to study distribution of native chromatin loci within intact mitotic chromosomes in cells isolated from *Drosophila* embryos. Our results reveal a higher than expected degree of organization, suggesting that the radial distribution of specific chromatin loci are non-uniform in fly mitotic chromosomes. Actively transcribed sequences were found to localize at the periphery of chromosomes during mitosis as labeled by specific histone modifications in fixed cells or by MSL3-GFP in vivo, while silent chromatin occupied more internal positions.

## Results

### Detecting Specific MSL3-Binding Sites on the *Drosophila* X Chromosome

To visualize discreet, specific loci in live *Drosophila* cells, we took advantage of the observation that ∼80% of active X chromosome genes are clearly marked by DCC and only ∼1% of genes are free of DCC. DCC specifically binds a subset of genes on the euchromatic arm of the X chromosome in males and is necessary for about 2-fold up-regulation in expression levels through local modification of chromatin [27]. For live imaging of the DCC, a fly line was created carrying four copies of an MSL3-GFP fusion, marking specific MSL3-binding sites on the X chromosome with GFP, and two copies of a *Drosophila* H2A histone variant, His2AvD of the H2A.F/Z family, fused with mRFP [28] labeling total chromatin (Figure S1A). His2AvD is widely distributed in the genome. It is enriched in thousands of euchromatic bands and the heterochromatic chromocenter [29]. H2AvD, similar to H2A, associates less tightly with DNA in transcribed sequences. MSL3-GFP was fully functional as judged by transgenic rescue of *msl3* mutant males. The transgene was expressed from the native *msl3* promoter, and the presence of four copies did not cause ectopic staining as judged by the similarity to wild type MSL3 immunofluorescence staining patterns and the lack of cytoplasmic or nucleoplasmic background. To study the organization of the euchromatic arm of the X chromosome we focused on neuroblasts (NBs) [30]: diploid, dividing, and easily identifiable cells in dissected brains of 3^rd^ instar larvae (Figure S1 B) and primary cultures isolated from 5–6-h-old embryos (Figure S1C). After isolation, primary cultures and tissues survived for up to 4 h of imaging without a change in medium, going through several divisions with normal cytokinesis and producing normal progeny. Embryonic cultures were isolated and deposited in a drop of Chan-Gehring medium on a cover slip glued to a microscope slide and sealed with enough air. The identity of NBs was confirmed by their size, 8–12 µm, the presence of smaller cells around them, and antibody staining against Dpn [31].

Fixed samples were imaged with wide-field deconvolution microscopy and the recently implemented structured illumination microscopy (SIM) technique. SIM has doubled resolution in X, Y, and Z, as compared to conventional wide-field microscopy. The excitation illumination dose was minimized through the use of optimized and dedicated emission filter sets: DAPI (460±25 nm), FITC (515±15 nm), and RHOD (590±15 nm). The 3D positions, orientations, and magnifications of signals imaged with FITC and RHOD emission channels varied systematically due to the differences in alignment of cameras and the chromatic aberration. In multi-color images, these channels were aligned using parameters calculated from multi-color micro-bead Z-stacks for wide-field microscopy or SIM (described in Supporting Methods in Text S1). The values of parameters were found as an optimization problem solution minimizing differences between the positions of the beads in different channels (Text S1 and Figure S2).

### Dosage Compensated Sequences Target to the Periphery of Live and Fixed Mitotic and Interphase Chromosomes

**Figure 1:**
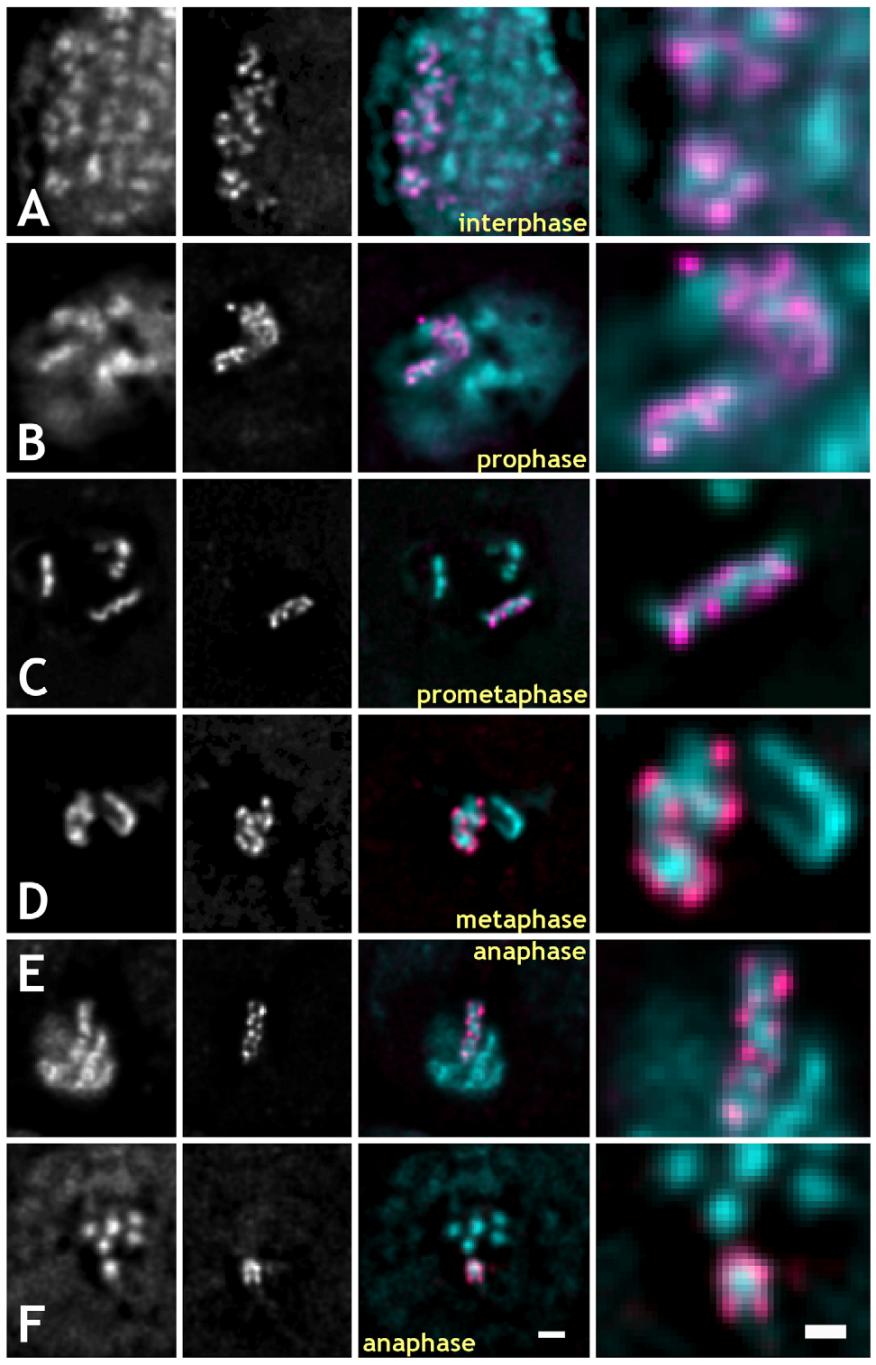
MSL3-GFP targets to the periphery of chromosomes in live embryonic cultured cells expressing MSL3-GFP and His2AvDmRFP1 at different stages of the cell cycle. For all pseudo-colored images: RFP1, cyan; GFP, magenta. (A–F) Rows from top to bottom: interphase, prophase, prometaphase, metaphase, anaphase (side view), and anaphase (cross-section view). Columns from left to right: His2AvDmRFP1-labeled chromatin, MSL3-GFP, superimposed mRFP1 (cyan) and GFP (magenta) signals, 3-fold higher magnification of the labeled X chromosome arm. All images were deconvolved. Bars: 1 µm – (F, third column), G, H, I; 0.5 µm – (F, last column).

In live embryos, bright foci of MSL3-GFP binding scattered uniformly over the entire nucleus are first detected late in cell cycle 14, during interphase before the first asynchronous division in the cellularized embryo. Within a single cell cycle, scattered MSL3-marked foci relocate into a relatively compact nuclear sub-region, ∼10%–30% of the nucleus area in projection (Figure 1A). The signal retains its specificity and brightness during the entire cell cycle throughout development, making it possible to use embryonic and larval brain NBs for imaging of chromatin loci specific to male X chromosomes (Video S1).

**Figure 2:**
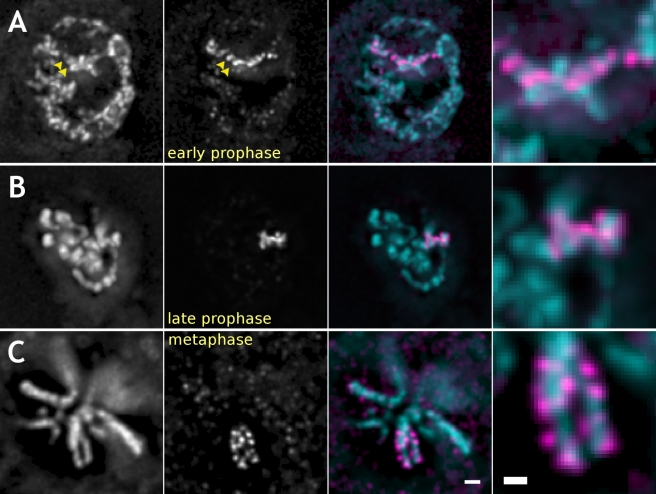
Active genes localize to the periphery of mitotic chromosomes in mitosis starting late prophase in fixed cells of embryonic cultures. Columns: first – total DNA labeled by His2AvDmRFP1; second – MSL3-GFP signal; third – superimposed, pseudo-colored GFP, and RFP channels; right – 3-fold higher magnification of the labeled region from the third column. In embryonic cultured cells expressing MSL3-GFP and His2AvDmRFP1 and fixed in early prophase (A), GFP and mRFP1 signals do not overlap. Rather, they complement each other (shown with arrowheads) within the partially condensed prophase chromosome arm, an extended structure 3–5 µm long and ∼200 nm wide. At early prophase, MSL3-GFP signal is often found to run across the entire width of condensing chromosomes. In addition, domains of MSL3-GFP signal are sandwiched between the domains of His2AvDmRFP1 signal without overlapping. In contrast, late prophase (B) and metaphase (C) distributions of MSL3-GFP are peripheral and locate to the periphery of the condensed chromosomal arms in all observed cells. Bars: 1 µm – first three columns and 500 nm – right column.

To study localization of MSL3-GFP-marked specific sites on X chromosomes, live primary embryonic cultures isolated from the fly line expressing both MSL3-GFP and His2AvDmRFP1 were imaged with wide-field microscopy. Fast Z-stacks, 20–30 sections per second, were collected, followed by deconvolution with a measured point-spread function (PSF). In embryonic cultures, all interphase and late prophase through telophase cells showed peripheral localization of MSL3-GFP with respect to adjacent chromatin (Figure 1A–F). An example Z-stack of a fixed metaphase NB expressing MSL3-GFP and His2AvDmRFP1 is shown in Video S2. In early prophase, MSL3-GFP domains were often found sandwiched between bulk chromatin domains (Figures 1B and 2A) running the entire width of the chromosome. In live anaphase chromosomes of dissected 3^rd^ instar larval brains imaged as Z-projections, MSL3-GFP was also peripheral in all observed cases. On all anaphase chromosomes, the MSL3-GFP pattern looked like two approximately parallel, ∼1–3 µm long, segments separated by ∼200–300 nm (Figure 1E). Figure 1F shows a cross-section of a live anaphase chromatid where the mRFP1-marked chromatin signal is clearly inside the peripheral GFP signal. The majority of foci of interphase MSL3-GFP, similarly to mitosis, localized to the periphery of chromatin domains marked by His2AvDmRFP1 with occasional partial overlap (Figure 1A). A similar organization of chromatin was observed in embryonic cultures fixed after isolation. This is very different from polytene chromosomes in which MSL3-GFP and His2AvDmRFP1 bands demonstrated, although not perfectly, a high degree of co-localization (1A). Immunostaining of fixed embryonic cultures for MSL2 (another DCC component) confirmed our finding that in X chromosomes, specific DCC-binding sites marked by MSL3-GFP target to the edges of compact chromatin domains in interphase or at the chromatid surface in mitosis (Figures S3 and S4 and Video S3).

In live or fixed interphase cells expressing MSL3-GFP and His2AvDmRFP1, the MSL3-GFP labeled X chromosome arm was a diffuse “cloud” of 20–30 foci of different intensity occupying 10%–30% of the nucleus area in projection, or 3–5 µm in linear dimensions. These foci were found at the periphery of condensed chromatin domains labeled with His2AvDmRFP1. In early prophase cells, the MSL3-GFP labeled condensing arm is a relatively compact structure, ∼4–6 µm long and ∼1 µm thick (Figure 2A). Interestingly, the MSL3-GFP signal was found not at the periphery of early prophase chromosomes but across their entire width between the bulk condensed chromatin regions (arrowheads in Figure 2A). The early prophase MSL3-GFP signal was complementary to the bulk chromatin domains marked by His2AvDmRFP1 but not peripheral. Transition from the internal to peripheral localization of MSL3-GFP occurred between early and late prophase (Figure 2A and B) before segregation of sister chromatids became apparent. After segregation at metaphase, MSL3-GFP stayed peripheral on both chromatids (Figure 2C). MSL3-GFP is found inside condensing early prophase chromosomes; however, starting from late prophase, MSL3-GFP signal is found only at the periphery of mitotic chromosomes in live or fixed cells.

**Figure 3:**
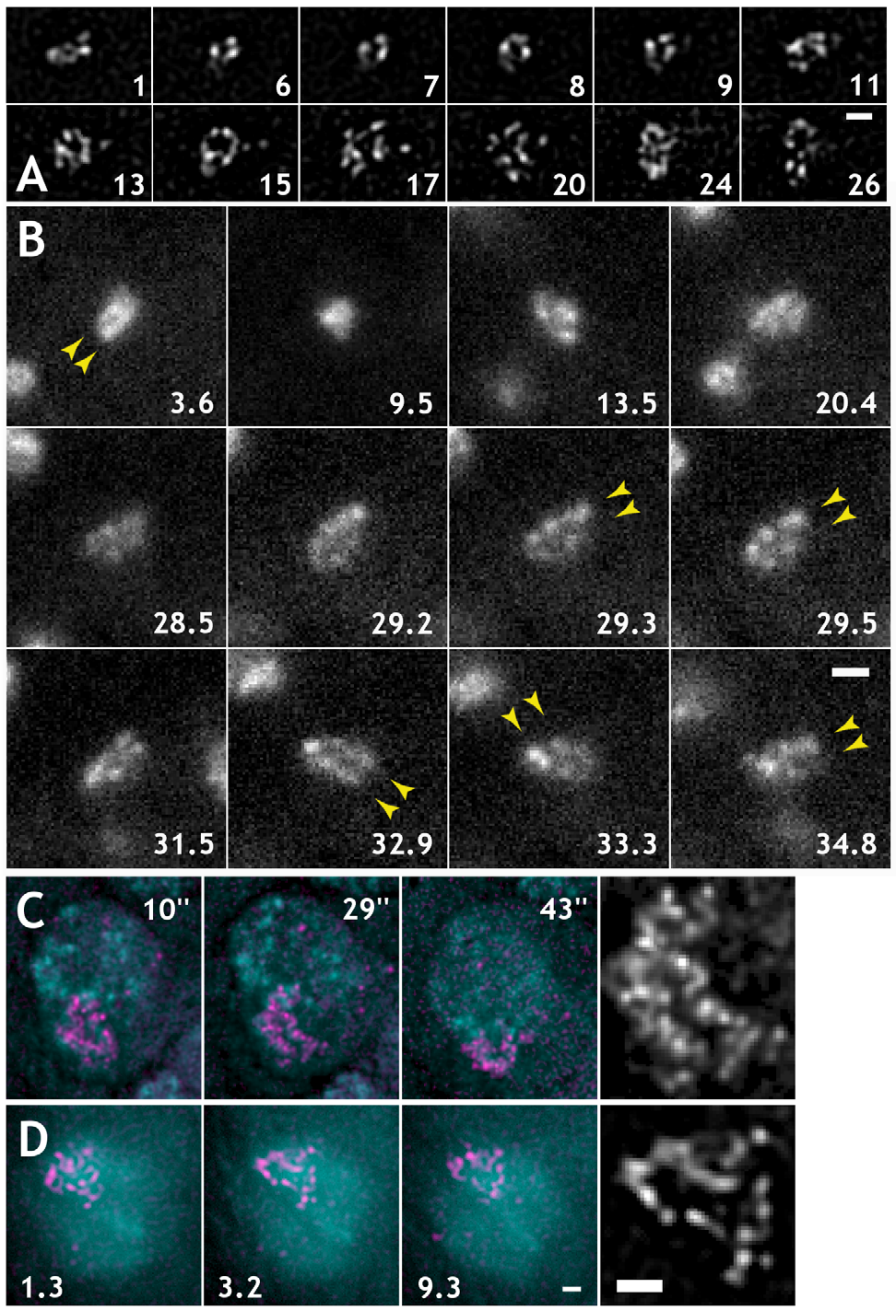
MSL3-GFP, peripheral during anaphase, remains mostly peripheral during decondensation. (A, B) MSL3-GFP does not redistribute during post-mitotic decondensation and interphase. The numbers represent minutes after beginning of observation. The first images of (A) and (B) are captured in mid-anaphase: peripheral MSL3-GFP is shown. (A) Anaphase to interphase dynamics of MSL3-GFP labeled X chromosomes in live 3^rd^ instar larval brains. (B) Anaphase to interphase dynamics of MSL3-GFP labeled X chromosomes in live embryonic cultures. Arrowheads point to apparent halves of MSL3-GFP signal, with an area inside devoid of MSL3-GFP. In many cells of live embryonic cultures (C) and tissues (D), MSL3-GFP labeled chromatin loci in interphase NBs form a typical pattern: a rim of MSL3-GFP signal around a region with no or little GFP signal. Gray-scale images in (C) and (D) are 3-fold magnifications of center colored panels. Numbers represent seconds (C) and minutes (D) after beginning of observation. All images, except for (B), were deconvolved. Bars: 1 µm.

DCC localization to the periphery of condensed chromosomes suggests that mitotic chromosome organization is correlated with its function. However, an alternative explanation could be that MSL complex reorganizes during mitosis to be released from condensed regions, and then re-binds after decondensation. Despite the small size of MSL3-GFP (∼100 kDa), the fully assembled MSL complex is thought to be at least 1 MDa. If the accessibility of chromatin targets inside condensed mitotic chromatin is limited, DCC complexes displaced during mitotic condensation would need to re-assemble at their proper targets during decondensation. We found no evidence for significant or noticeable redistribution or loss of MSL3-GFP during mitotic condensation in agreement with the extremely stable association of MSL2 with its targets both during interphase and mitosis [32]. The dynamics of the MSL3-GFP-labeled chromatin regions could be followed during mitotic condensation. In our Video S1, we show an example of progression from smaller faint speckles scattered over a large area to bright and compact foci on mitotic chromosomes through condensation and fusion. To further analyze possible redistribution of MSL complexes over the cell cycle, anaphase cells of embryonic cultures or larval brains were imaged for extended periods of time after cytokinesis to allow decondensation of chromatin and cell cycle-related redistribution of nuclear proteins. Dividing cells of embryonic cultures (Figure 3A) or in dissected 3^rd^ instar larval brains (Figure 3B) were imaged with fast Z-stacks or Z-projections. During post-mitotic decondensation, MSL3-GFP-marked regions peripheral in anaphase moved outwards during decondensation, expanding 2–3-fold in the area over a period of 10–20 min, shown in time-series in Figure 3A and B and Video S4. The unlabeled regions of chromosomes, internal during anaphase, remain unlabeled for up to 30 min or more than a half cell cycle duration, when accessibility of the euchromatic X chromosome arm should no longer be an issue. For embryonic cultures expressing MSL3-GFP and His2AvDmRFP1, it takes 4 to 6 min for a dividing cultured NB to proceed from mid-anaphase to a state in G1 with a round nucleus, fully decondensed chromatin, and reformed nucleoli as judged by His2AvDmRFP1 staining. The increase in the area of the MSL3-GFP-marked region occurred exclusively through expansion of chromosome arm, not redistribution or *de novo* binding of MSL3-GFP. We conclude that the regions devoid of MSL3-GFP signal in mitotic chromosomes do not appear to be targets of MSL complex upon decondensation.

Another line of evidence for limited re-distribution comes from the low, little changing cytoplasmic MSL3-GFP background over the entire cell cycle. The background intensity of MSL3-GFP in mitotic cytoplasm is comparable to the uniform background intensity outside the cells created by out-of-focus and scattered light. For a metaphase cell, the outside background was 15–35 units (mean 24), the cytoplasm was 25–55 (mean 38), compared to the labeled X chromosome arm—125–525 (mean 305). In interphase, the nuclear and cytoplasmic background intensity of MSL3-GFP is comparable or higher than in mitosis. This suggests that in mitosis the majority of MSL3-GFP is divided between daughter cells by traveling on chromosomes and that there is minimal loss of MSL3-GFP from chromatin targets during mitotic condensation.

The MSL3-GFP labeled loci often surround an area of no or low GFP signal in live embryos of dissected tissues (Figure 3C and D and Figure S1C and B, respectively), despite their extremely dynamic behavior in interphase. In the majority of interphase nuclei, MSL3-GFP labeled regions demonstrate apparent reduction of GFP signal intensity in the middle of the labeled arm as shown in Figure 3C and D. These observations are consistent with the previous FISH studies of fixed tissue culture cells [33],[34].

The distribution of MSL3-GFP during mitosis or interphase is clearly distinct from the binding patterns of perichromosomal layer proteins, such as Ki-67 and nucleophosmin, which are associated with the outer surface of chromosomes in mitosis and populate the vicinity of chromosomes in the form of granules and fibrils [35]. MSL3-GFP and components of the DCC remain bound exclusively to chromatin locations of the euchromatic X chromosome arms at all stages of the cell cycle. In contrast, the proteins of the perichromosomal layer cover the periphery of all mitotic chromosomes over their entire length, except centromeres, from prophase to telophase. In interphase, the proteins of the perichromosomal layer are found in nucleoplasm and cytoplasm with preferential accumulation at nucleoli.

### Histone Modifications Are Distributed Non-Uniformly in Mitotic Chromosomes

**Figure 4:**
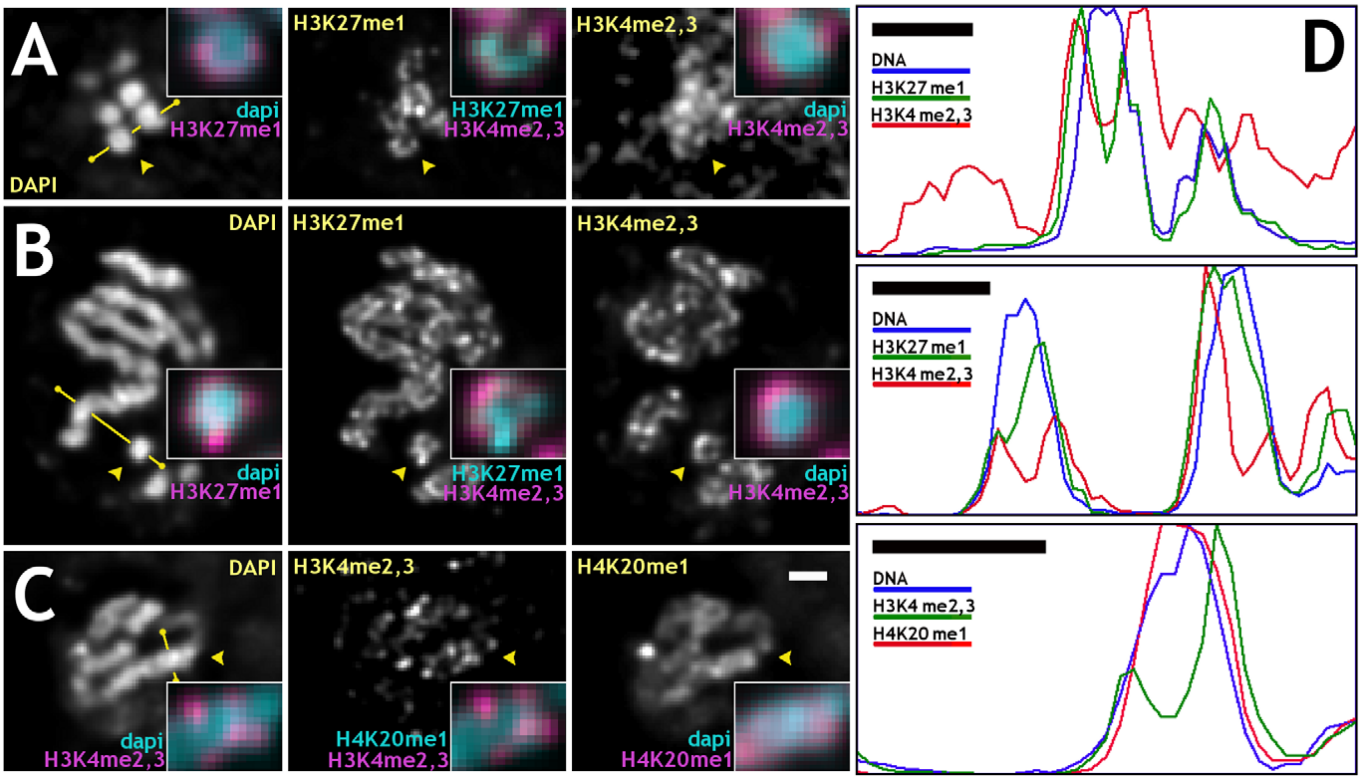
The density of specific histone modifications varies across metaphase and anaphase chromosomes. (A–C) Double antibody staining against specific histone modifications in fixed embryonic cultures is radially non-uniform in mitotic chromosomes. In (A) and (B), examples are shown of cells stained with DAPI for DNA (left images), anti-H3K27me1 (center images), and anti-H3K4me2,3 (right images) antibodies, (C) DAPI (left), anti-H3K4me2,3 (middle), and anti-H4K20me1 (right) antibodies. (A) An optical cross-section of anaphase chromosomes oriented along the optical axis; (B) side view of metaphase chromosomes; (C) side view of anaphase chromosomes. Arrowheads point to regions magnified 2.5-fold in insets. Insets: (A,B) DAPI (cyan) and anti-H3K27me1 (magenta) – left column; anti-H3K27me1 (cyan) and anti-H3K4me2,3 (magenta) – middle column; DAPI (cyan) and anti-H3K4me2,3 (magenta) – right column. (C) DAPI (cyan) and anti-H3K4me2,3 (magenta) – left column; anti-H4K20me1 (cyan) and anti-H3K4me2,3 (magenta) – middle column; DAPI (cyan) and anti-H4K20me1 (magenta) – right column. (D) Profiles for antibody stained chromosomes along lines marked by yellow segments in (A–C) in the same order. In interphase chromosomes, anti-MSL2 and anti-H3K4me2,3 signals marking active loci occupy regions spatially distinct from anti-H3K27me1 and anti-H4K20me1, demonstrating that tightly compacted mitotic chromatin is accessible to antibodies. Images were deconvolved. Bars: 1 µm – (C, D).

Dosage compensated genes represent a large subset of active male X chromosome genes with the transcription rate up-regulated about 2-fold as compared to non-compensated genes [27],[36]. *Drosophila* chromatin regions of different transcription states are known to be marked with specific histone modifications. Patterns of covalent core histone modifications are established in *Drosophila* embryos by cell cycle 15. We chose di- and trimethylation of lysine 4 on histone H3 (H3K4me2,3) as a marker for active euchromatin, and H3K27me1 and H4K20me1 as markers for silent, non-coding regions in euchromatic chromosomal arms for immunofluorescence. Double-antibody staining served two purposes: (a) to study the distribution of actively transcribed sequences over the entire X chromosome and autosomes, and (b) to study the distribution of histone modifications specific to different transcription states. In mitotic chromosomes, anti-H3K27me1 and anti-H4K20me1 antibodies were found to stain all chromosomes uniformly along their lengths, on both euchromatic and heterochromatic arms. In contrast, anti-H3K4me2,3 was limited to euchromatic arms of mitotic chromosomes, an indication of the specificity of the antibodies. We found that immunofluorescence signals from anti-H3K27me1 and anti-H4K20me1 were consistently found at more internal locations on chromosomes compared to the anti-H3K4me2,3, as summarized in Figure 4A–C for mitotic cells. Line profiles were used for better visualization of non-uniform distribution of different antibodies (Figure 4D). Anti-H3K4me2,3, as well as anti-MSL2, antibodies always stained the periphery of chromosomes, with a significant fraction of the signal found outside the visible DAPI signal. This is different from anti-H3K27me1 and anti-H4K20me1 signals, which were found mostly inside chromosomes with no or little signal extending into DAPI-free regions. In multiple examples, anti-H4K20me1 staining coincides with the DAPI signal uniformly staining chromosomes (Figure 4C). Anti-H3K27me1 signal stains, though not uniformly, the entire width of chromosomes or often with visible reduction of signal at chromosomal cores (Figure 4A and B). A more internal and uniform signal of anti-H4K20me1 antibody compared to anti-H3K27me1 is consistent with localization of H4K20me1 to relatively more condensed regions of genome (chromocenter and few euchromatic bands in polytene chromosomes). To support these observations, the widths and distributions of fluorescent labels after immunofluorescence staining with different antibodies or in live cells were measured (Table 1). The widths of chromosomes, immunostaining, and MSL3-GFP signals were measured as FWHM in averaged profiles. It is seen that anti-H3K4me2,3 and live MSL3-GFP signals localize to the periphery of mitotic chromosomes and are depleted at the cores of mitotic chromosomes relatively to the anti-H3K27me1 and anti-H4K20me1 signals, which show less depletion at the core (Figure S5). This is additional evidence that active coding sequences target to the periphery of chromosomes in mitosis. Hypothesis testing of the null hypothesis of equal means was done for each pair of data sets for different labeling methods and summarized in Table S1. In support of our observations and measurements, the null hypotheses of equal means were rejected for the following pairs: H3K4me2,3 and H4K20me1; H3K4me2,3 and H3K27me1; H4K20me1 and MSL3-GFP; MSL3-GFP and H3K27me1. Rejection of the null hypothesis of equal means is not supported for the following pairs: H3K4me2,3 and MSL3-GFP; H3K27me1 and H4K20me1. This is in agreement with similarity in the distributions of the signals and their widths. All primary antibodies used for immunofluorescence were of the monomeric IgG type, and secondary antibodies were F(ab')_2_ fragments labeled with a fluorophore.

**Table 1:** Immunofluorescence staining against specific histone modifications and live MSL3-GFP signal have different width and distribution on mitotic chromosomes.

	Anti-H3K4me2,3	Anti-H4K20me1	Anti-H3K27me1	Live MSL3-GFP
Number of chromosomes	21	20	18	15
Mean chromosome width, nm	467 (66)	468 (58)	463 (79)	482 (73)
Mean IF staining width, nm	632 (91)	499 (67)	533 (108)	633 (68)
Mean ratio: “IF”/“DNA”	1.34 (0.16)	1.07 (0.07)	1.15 (0.15)	1.33 (0.17)
Two peak separation, nm	358 (50)	276 (50)	308 (65)	353 (51)

Rows from top to bottom: number of chromosomes measured for each data set; mean chromosome width after DAPI staining for the antibody stained chromosomes or for His2AvDmRFP1-labeling for live chromosomes in MSL3-GFP-expressing tissues; mean immunofluorescence (IF) staining width (the first three columns) or MSL3-GFP signal width (the rightmost column) for live cells; individually normalized and averaged widths of antibody or MSL3-GFP labels, “IF” is an antibody staining width, “DNA” is a chromosome width; average separation of centers of peripheral antibody or MSL3-GFP signals across the chromosome width (summarized in Figure S5 and more details can be found in Text S1).

**Figure 5:**
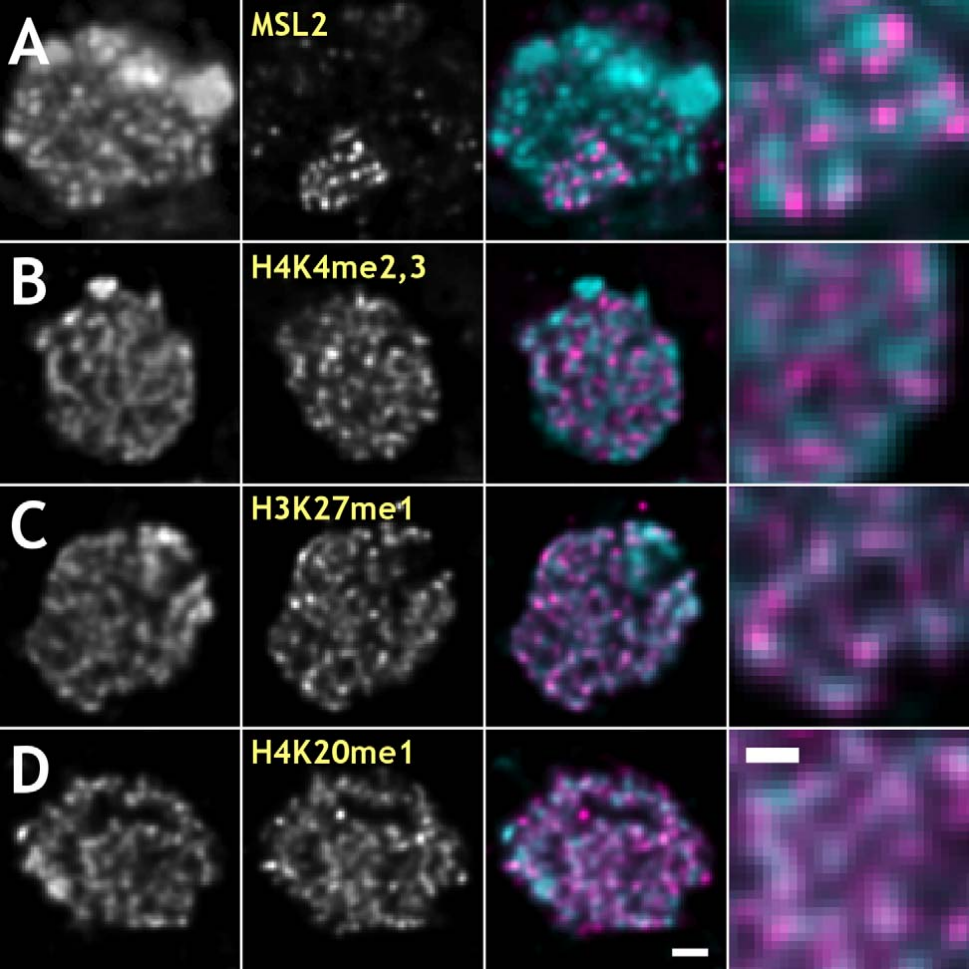
In fixed, antibody stained interphase cells from embryonic cultures anti-MSL2 (A) and anti-H3K4me2,3 (B) signals localize to the periphery of chromatin domains as detected by DAPI staining, while anti-H3K27me1 (C) and anti-H4K20me1 (D) signals largely overlap with chromatin. Columns from left to right: DAPI for DNA, antibody staining, pseudo-colored superimposition of DAPI (cyan) and immunofluorescence (magenta) signals, 3-fold expanded region of the superimposition. Bars: 1 µm – (D) (third column); 0.5 µm – (D) (right column).

In fixed interphase cells, actively transcribed sequences labeled with anti-MSL2 or anti-H3K4me2,3 (pseudo-colored magenta) are found outside chromatin regions labeled with DAPI and were complementary to them (Figure 5A and B, respectively). In contrast, anti-H3K27me1- and anti-H4K20me1-labeled silent chromatin was a subset of DAPI stained chromatin largely overlapping with it (Figure 5C and D, respectively).

To investigate the accessibility of mitotic chromatin to antibodies, we stained fixed mitotic cells with antibodies to Barren and to CID-GFP, both known to be buried inside mitotic chromosomes. Barren, a member of the kleisin family and an ortholog of human CAP-H, binds to the head domains of the SMC heterodimer in a complex with two other non-SMC subunits [37]. As a part of the condensin complex, Barren accumulates at the core regions of mitotic chromosomes starting in prophase through metaphase. Anti-barren [38] immunofluorescence signal was found at the central core regions of the DAPI stained chromosomes imaged with wide-field or SIM (Figure S6A and B, respectively), similar to live distributions (Y. Strukov, unpublished results). CID, a *Drosophila* ortholog of human CENP-A, is an H3-like protein that replaces canonical H3 in all eukaryotic centromeres [39]. It was proposed that CID is the epigenetic mark and a foundation for fly centromeres and localizes beneath the kinetochore with some overlap with the inner kinetochore [40]. Anti-GFP immunofluorescence of CID-GFP embryonic cultures gave a uniform labeling over all centromeres in mitotic cells (the same as in live cells), demonstrating accessibility of chromatin buried by the kinetochore structures to antibodies under our fixation conditions (Figure S6C and D). Centromeres of CID-GFP expressing cells had the same size and shapes irrespective of the imaging modality: live imaged with wide-field deconvolution, antibody-stained imaged with wide-field deconvolution, or antibody-stained imaged with SIM (Figure S6E), demonstrated by intensity profiles (Figure S6F).

## Materials and Methods

### Fly Lines

A 5.5-kb BamHI genomic fragment containing the promoter and open reading frame of the *msl3* gene [41] was subcloned from cosmid msl3-5-1 (AE003560.1 position 60298–92793) into the pBluescript II SK(−) vector [12]. A blunted NcoI/NotI fragment containing eGFP from the pEGFP-N1 vector (Clontech) was subcloned in-frame into the CelII blunted pBS-*msl3* construct. The resulting MSL3-GFP BamHI fragment was subcloned into pCaSpeR3 to make the final MSL3-GFP–pCaSpeR3 construct. Several independent transgenic lines were produced by P-element-mediated transformation [42]. Two independent lines, one with the transgene on the 2^nd^ and one with it on the 3^rd^ chromosome, were crossed to generate a stock with homozygous MSL3-GFP on both chromosomes (4 copies). Fly line msl3-gfp, His2AvDmRFP1; msl3-gfp was created by recombination of msl3-gfp; msl3-gfp with a His2AvDmRFP1 transgenic line [28] for dual-color live experiments; CID-GFP line was generated in the lab of S. Henikoff (FHCRC).

### Sample Preparation and Immunostaining

Embryonic cultures were isolated according to a previously published protocol [30]. The fixation and immunostaining protocols were adapted from [43]. Antibodies were from Abcam (Cambridge, MA): rabbit polyclonal anti-H4K20me1 (ab9048), and anti-GFP (ab290), mouse monoclonal anti-H3K4me2,3 (ab6000), and Upstate Scientific: rabbit anti-H3K27me1 (07-448), all monomeric IgG. Rabbit anti-MSL2 antibody was generated in the Kuroda laboratory [44]. Goat anti-mouse or anti-rabbit secondary antibodies were from Molecular Probes: A-11017, A-11018, A-11070, A-11071.

### Microscopy and Image Processing

Live, wide-field optical sectioning and SIM were done on a custom-made inverted microscope [26] supplied with a set of excitation lasers and cooled back-thinned CCD cameras (Andor). Deconvolution and SIM reconstruction were done using a measured PSF [45]. Image processing and computations were done using Priism, Python (align.py and simplex.py), and Octave scripts available upon request (contact YGS: yu.strukov@gmail.com).

## Discussion

Our investigation has provided insights into mitotic chromatin organization and its connection to chromatin function. We observed several novel features of mitotic chromosomes in *Drosophila* cells. First, MSL3-binding sites, specific to the X chromosome, target to the periphery of mitotic chromosomes. Second, spatial distribution of chromatin loci within mitotic chromosomes was correlated with their functional properties judged by the core histone modifications. Third, during late prophase-to-anaphase condensation of chromatids, active sequences remain peripheral, suggesting rearrangement of chromatin within prophase chromatids and arguing against simple coiling of prophase chromatids during condensation. Although several investigations have been undertaken in the past, to our knowledge, ours is the first when native chromosomes in live cells have been studied for localization of specific DNA sequences at high resolution.

All imaging systems with multi-color capability are known to suffer from chromatic aberrations and offsets in translation, rotation, and magnifications between color channels. For interpretation of multi-color data sets, correct superimposition of different color channels was therefore critical. To exclude the influence of the chromatic aberration of the objective lens, variations in the CCD camera specifications, and differences in optics between different emission channels, control multi-color data sets were collected with multi-color micro-beads, separately for wide-field or SIM and compensated to a sub-pixel accuracy using custom software (Supporting Methods in Text S1 and Figure S2). Based on these measurements, we could conclude that peripheral localization of specific sequences marked by DCC or core histone modifications was not due to chromatic aberration or differences in the optics of individual channels.

MSL complex specifically marking dosage compensated genes in *Drosophila* provides a means for visualization of a functionally distinct fraction of the genome. The 22 Mbp X chromosome arm, predicted to contain about a thousand active genes, is ∼5% of the total DNA content of a diploid male *Drosophila* cell. There is evidence that ∼80% of active genes on the X chromosome are bound by DCC and less than 1% are clearly free of it, the rest of the genes are bound by intermediate amounts of MSL complex [46]. MSL3 binds preferentially to 3′ ends of transcribed regions of most active genes. In larval polytene chromosomes, MSL complex binds to gene-rich interbands, complementary to DAPI-stained bands, and co-localizes with H4 acetylated at lysine 16, a specific mark for open, transcriptionally active chromatin in general, both in vivo and in vitro [47]. Little is known about MSL binding dynamics, however it is possible that MSL localization is established at most active gene clusters early in development and maintained in a relatively static pattern throughout development [47]. However, blocking transcription can inhibit MSL complex binding at a transgenic target, suggesting that the MSL pattern is not completely static [48]. There is a possibility that MSL complex changes its localization in condensing chromosomes, but the fact that it looks similar to H3K4me2,3 (and different from H4K20me1) is strong evidence that it has stayed with the active regions. Our results support the possibility that little or no change in MSL targeting occurs during mitosis. We have shown that there is no appreciable dissociation and relocation of MSL3-GFP from chromosome targets to other cellular compartments at the beginning or during mitosis by measuring the cytoplasmic and nucleoplasmic backgrounds of MSL3-GFP. The entire population of MSL3-GFP remains bound to the X chromosome. No significant new binding of MSL3-GFP to the chromosome targets was observed during post-mitotic decondensation (Figure 3A and B). This is consistent with a number of earlier findings: MSL complex does not bind at significant levels to non-coding sequences and more than 90% of MSL complexes are found within genes [13],[14]; there has been found little [12],[49] or no [50] variation in the binding levels at specific loci during development. Redistribution of MSL3-GFP within the X chromosome by fast dissociation from the interphase set of targets to a new non-overlapping mitotic set would be hard to imagine for several reasons. First, most genes on the X chromosome in interphase are bound by MSL complexes [16] and there is no binding of MSL complex to non-coding DNA at the levels sufficient to accommodate the entire pool of DCC. This suggests a great deal of overlap between interphase and mitotic MSL complex binding sites. Second, available data on the dynamics of MSL binding to its targets show that the time-scale for MSL targeting is on the order of hours [50], which is not consistent with very fast cell mitoses and cycles of chromatin condensation-decondensation in developing Drosophila tissues. Testing mitotic distributions of MSL3 with ChIP at high resolution is currently unfeasible due to the technically challenging procedure of isolating sufficient quantities of purified mitotic cells from dissected tissues and embryonic cultures from Drosophila. At the resolution of fluorescence microscopy analysis we have demonstrated that binding of MSL3-GFP is not cell-cycle stage specific. We chose His2AvDmRFP1 as a contrasting fluorescent marker because it is found in the heterochromatic chromocenter, on transcribed and non-transcribed genes and in non-coding euchromatin [29].

The current concepts of nuclear organization suggest a relationship between the activity of chromatin loci and their positions within nuclei or interphase chromosomes [51]. However, whether this principle can be extended to also include live and fixed mitotic chromosomes has not been investigated. The question of whether antibodies faithfully represent the imaged features is always important. There are two aspects to this problem: first, uniform accessibility of chromosomal epitopes; and second, the finite size of the antibody complex with a potential to change the size and shape of sampled features. We argue that with our sample preparation and staining techniques mitotic chromatin was available for antibodies. Anti-barren staining produced a continuous axial pattern indicating that internal regions of chromosomes were uniformly accessible. Anti-GFP antibody staining of embryonic cultures isolated from CID-GFP expressing fly embryos gave the patterns of centromere labeling with similar shapes and sizes as in live cells. Consistent with our observations are previous studies of centromere and kinetochore organization where both structures were shown to be accessible after formaldehyde fixation to various antibodies raised against different centromeric or kinetochore proteins [52],[53]. It has been demonstrated that in live cells, proteins with molecular dimensions in the size range of components of the transcription machinery (several hundred kDa) can diffuse freely inside condensed chromatin domains [54]. The mass of individual IgG molecules is ∼150 kDa, and the size of the primary and secondary fluorophore-labeled antibody complex has been reported to be about 20 nm [55], which makes it small enough to faithfully reflect the features of imaged objects at a resolution of about 200 nm used for immunofluorescence experiments.

**Figure 6:**
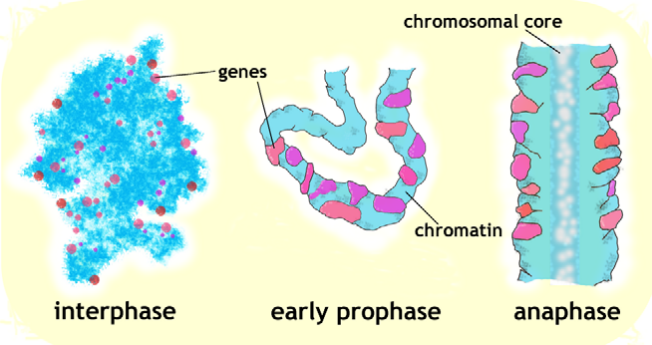
Formation of a radially non-uniform anaphase chromosome. Large-scale re-arrangements inside 100–200 nm wide early prophase fibers (in which MSL3-GFP signals are often found throughout the entire chromosome width) result in peripheral localization of active sequences in ∼500 nm wide metaphase and anaphase chromatids.

Using both immunofluorescence of fixed cells and live observations we showed that MSL3-GFP stays peripheral from late prophase to telophase in the same cell arguing against hierarchical coiling condensation [1]. If consecutive coiling is involved, the large-scale fibers have to have persistence length on the order of their thickness, and simultaneously, intra-fiber rearrangements have to be involved to keep the genes at the periphery of the X chromosome as mitotic condensation progresses. Our working model is shown in Figure 6: patches of chromatin carrying active genes, spanning the entire width of chromosomes at early prophase, become peripheral from late prophase through telophase as a result of large-scale rearrangements within condensing chromosomes.

An interesting explanation for why active DNA sequences are found at the periphery of mitotic chromosomes comes from biochemical studies of the “bookmarking” mechanism that helps cells remember which genes were active before mitosis. It was reported that active promoters/genes remain bound by a transcription factor TFIID in mitosis [56] and may escape the condensin complexes action through recruitment of the TBP-PP2A mitotic complex [57]. Transcribed sequences show more mitotic TBP binding than silent DNA. TBP interacts with the condensin I subunit CAP-G and condensin inhibitor phosphatase PP2A during mitosis at many chromosomal sites active before mitosis.

Our findings are consistent with the results of a number of earlier studies concentrated on the localization of specific DNA sequences or sequences of specific properties on mitotic chromosomes. However, our conclusions are based on observations of native chromatin loci in the context of unperturbed chromosomes. Specifically stained AT-rich DNA sequences in Munjac chromosomes formed a full-diameter coil at gene-poor regions and uncoiled in gene-rich regions staying at the core [7]. In accordance with the radially non-uniform organization, the AT-rich sequences were at the core regions of the gene-rich bands, while the rest of DNA in the bands was more peripheral. Radially different and reproducible positions of specific sequences after FISH of salt-extracted isolated chromosomes was observed in agreement with the radial-loop model, with no indication, however, of their positions in native chromosomes [6]. Preferentially external lateral positions of specific sequences on mitotic chromosomes in mitotic spreads have also been reported after FISH [58]. However, the conclusions were not as convincing due to limited resolution in the images and FISH procedure-induced disturbance of native morphology. The degree of reproducibility of radial positions of stably transfected and gene amplified lac op repeats varied in different tissue culture cell lines, probably due to position effects [4],[59]. Lac op repeats could be found either at the core regions of chromosomes or throughout the width of chromosomes.

Reproducibility of positioning of specific sequences might be related to functional contributions of diverse classes of loci to the structure. Actively transcribed sequences may be spared a structural function or cannot be involved because of their specific protein composition or kinetic restrictions due to delay in condensation compared to silent or non-coding DNA. Alternatively, there could be a difference in degree of condensation or in its temporal sequence between peripheral and more central regions of mitotic chromosomes. Together, our results suggest novel structural features of mitotic chromosomes that can contribute to the understanding of mitotic condensation, with important implications for understanding the connection between chromatin organization and its epigenetic regulation.

## Supporting Information

Figure S1 **Examples of polytene chromosomes, embryos, and tissues from a transgenic fly line carrying MSL3-GFP and His2AvDmRFP1.** (A) Live polytene nuclei isolated from 3^rd^ instar larvae of yw; [w+ M3-GFP]; [w+ M3-GFP] line. Top row: His2AvDmRFP1 and MSL3-GFP channels, respectively; middle row: superimposed, pseudo-colored images, with a close-up of 3-fold higher magnification; bottom row: His2AvDmRFP1 and MSL3-GFP are largely, though not perfectly, co-localized. The arrowheads show two co-localized bands in both channels. (B) Live brain of 3^rd^ instar larvae expressing MSL3-GFP. GFP signal of many X chromosomes shows radially non-uniform organization with reduced intensity in the middle of the signal. (C) Live embryos during gastrulation, cell cycle 15 with compact MSL3-GFP signals. Bars: 5 µm – (A) (top row), (C); 2 µm – (A) (middle row).
(3.39 MB TIF)

Figure S2 **Digital, postimaging alignment of different color channels is necessary to exclude the contributions of chromatic aberrations, relative differences in CCD camera adjustments, such as translations, rotations and magnification, and variations in the optical paths of the color channels.** Shown are the FITC and RHOD channels before (A) and after (B) alignment: the RHOD channel was translated, rotated, and magnification compensated to match the FITC channel. Top panels show the XY projections of a 3D bead data set, bottom panels – XZ projections. Bar: 1 µm.
(0.31 MB TIF)

Figure S3 **Actively transcribed sequences target to the periphery of chromosomes at different stages of mitosis and at interphase in fixed, anti-MSL2 antibody stained cells of embryonic cultures isolated from Oregon R line and imaged with SIM.** Despite overlap between anti-MSL2 and DAPI signals, some MSL2 stayed outside the DAPI-labeled chromatin. For each row, (A) through (E), shown are from left to right DAPI, anti-MSL2, pseudo-colored DAPI (cyan) and anti-MSL2 (magenta) superimposed, and a 2.5-fold increased magnification of the antibody labeled chromosome arm. (A) interphase; (B) prometaphase; (C) metaphase; (D) in anaphase, the anti-MSL2 signal was 400–600 nm in diameter with the DAPI-stained chromatid diameter of 400–500 nm. (E) telophase. Bars: 1 µm – whole cell images; 0.5 µm – expanded regions.
(7.13 MB TIF)

Figure S4 **Stereo-pairs of anti-GFP stained, SIM-imaged (single sister chromatid) chromosomes in fixed cells isolated from MSL3-GFP expressing embryos.** Only the euchromatic arm of X chromosome is labeled: side view with telomeres at the bottom (left) and axial view with a staining-free channel within an anaphase chromatid (right). Bar: 0.5 µm.
(0.11 MB TIF)

Figure S5 **Immunofluorescence staining against different histone modifications and the MSL3-GFP signal have different widths and intensity distributions relative to chromosomal DNA.** The intensities of individual profiles in each group was normalized, then averaged and plotted to demonstrate differences both in the relative widths and signal distributions. Each individual profile was an average over a straight linear segment of a chromosomal arm 15 pixels or about 1200 nm long. Anti-H3K4me2,3 and live MSL3-GFP signals had equal widths, ∼630 nm (std 91 nm), pronounced depletion of the signal at the core, and well-separated and coinciding peaks of peripheral signals. Anti-H3K27me1 was narrower than the first two, 533 nm (std 108) and had barely resolved peripheral signals with almost no drop of the intensity at the core. Anti-H4K20me1 signal was 500 nm (std 67) wide and had no dip at the core, similar in the profile to DAPI staining and suggesting that it stained more internal regions of chromosomes compared to MSL3-GFP or the other antibody signals. Normalization of individual profiles by the chromosome width measured with DAPI or His2AvDmRFP1 signals produced similar averaged values.
(0.22 MB TIF)

Figure S6 **Mitotic chromatin is not refractory to immunofluorescence.** Wide-field imaged metaphase (A) and SIM-imaged anaphase (B) chromosomes stained with anti-barren antibodies. From left to right: DAPI, anti-barren antibody, pseudo-colored and superimposed DAPI (cyan) and anti-barren (magenta), 2.5-fold higher magnification of the superimposition. The dimensions and the shapes of the centromeres are comparable in live and fixed cells. (C) Live cells, from left to right: His2AvDmRFP1, cid-GFP, cid-GFP (magenta), and His2AvDmRFP1 (cyan) combined. (D) Fixed cells, from left to right: DAPI, anti-GFP antibody, anti-GFP antibody (magenta), and DAPI (cyan) combined. (E) The appearance and dimensions of centromeres do not depend on labeling and imaging methods. From left to right: cid-GFP imaged with wide-filed microscopy, anti-GFP antibody staining imaged with wide-filed microscopy (both expanded from panels C and D), and centromeres after anti-GFP antibody staining of a fixed cell imaged with SIM. All non-SIM images were deconvolved. (F) Intensity line profiles across centromere images with different modalities are comparable at FWHM. Bars: 0.5 µm – (A), (B), (D) (centromere images); 0.1 µm – line profiles in (F).
(0.94 MB TIF)

Table S1 **The results of hypothesis testing with the null hypothesis of pair-wise equal means and different alternative hypotheses are in agreement with the observed variations in the distribution of histone modifications across the chromosome width.** One-sided alternative hypothesis H_1_ was that the mean of the first data set was greater than the mean of the second data set in the same row. Two-sided alternative hypothesis H_1_ was that the mean of the first data set was less or greater than the mean of the second data set in the same row. The results of hypothesis testing support rejection of the null hypothesis of equal means for the following pairs: H3K4me2,3 and H4K20me1; H3K4me2,3 and H3K27me1; H4K20me1 and MSL3-GFP; MSL3-GFP and H3K27me1. Rejection of the null hypothesis of equal means is not supported for the following pairs: H3K4me2,3 and MSL3-GFP; H3K27me1 and H4K20me1. This is in agreement with our conclusions based on visual observations and examination of individual profiles.
(0.11 MB DOC)

Text S1 **Supporting text contains extra details and discussion of methods and supporting figures and videos.** Supporting information includes detailed protocols of preparation of *Drosophila* embryonic cultures and dissected tissues, fixation and immunostaining procedure, and fluorescence microscopy imaging and image processing.
(0.14 MB DOC)

Video S1 **Mitotic condensation of chromatin in embryonic cultures isolated from msl3GFP, His2AvDmRFP1; msl3GFP line.** Maximum intensity projections of 6 optical sections 0.5 µm apart; images were taken once every 40 s.
(0.16 MB AVI)

Video S2 **A Z-stack of fixed metaphase cell in embryonic culture isolated from msl3GFP, His2AvDmRFP1; msl3GFP line: optical sections are 150 nm apart.**
(0.13 MB AVI)

Video S3 **3D reconstruction of anti-GFP staining of fixed anaphase X chromosome of MSL3-GFP line imaged with SI.**
(0.33 MB AVI)

Video S4 **Live decondensation of MSL3-GFP labeled chromosome in larval brains from mid-anaphase through interphase: 178 time points, 10 s apart.**
(0.30 MB AVI)

## Abbreviations

DCC: dosage compensation complex
MSL: Male Specific Lethal
NB: neuroblast
PSF: point-spread function
SIM: structured illumination microscopy
