# Supplementary figures and images for: Evidence of Activity-Specific, Radial Organization of Mitotic Chromosomes in Drosophila
Source: PLoS Biol. 2011 Jan 11;9(1):e1000574. doi: 10.1371/journal.pbio.1000574 (PMC3019107; doi:10.1371/journal.pbio.1000574)

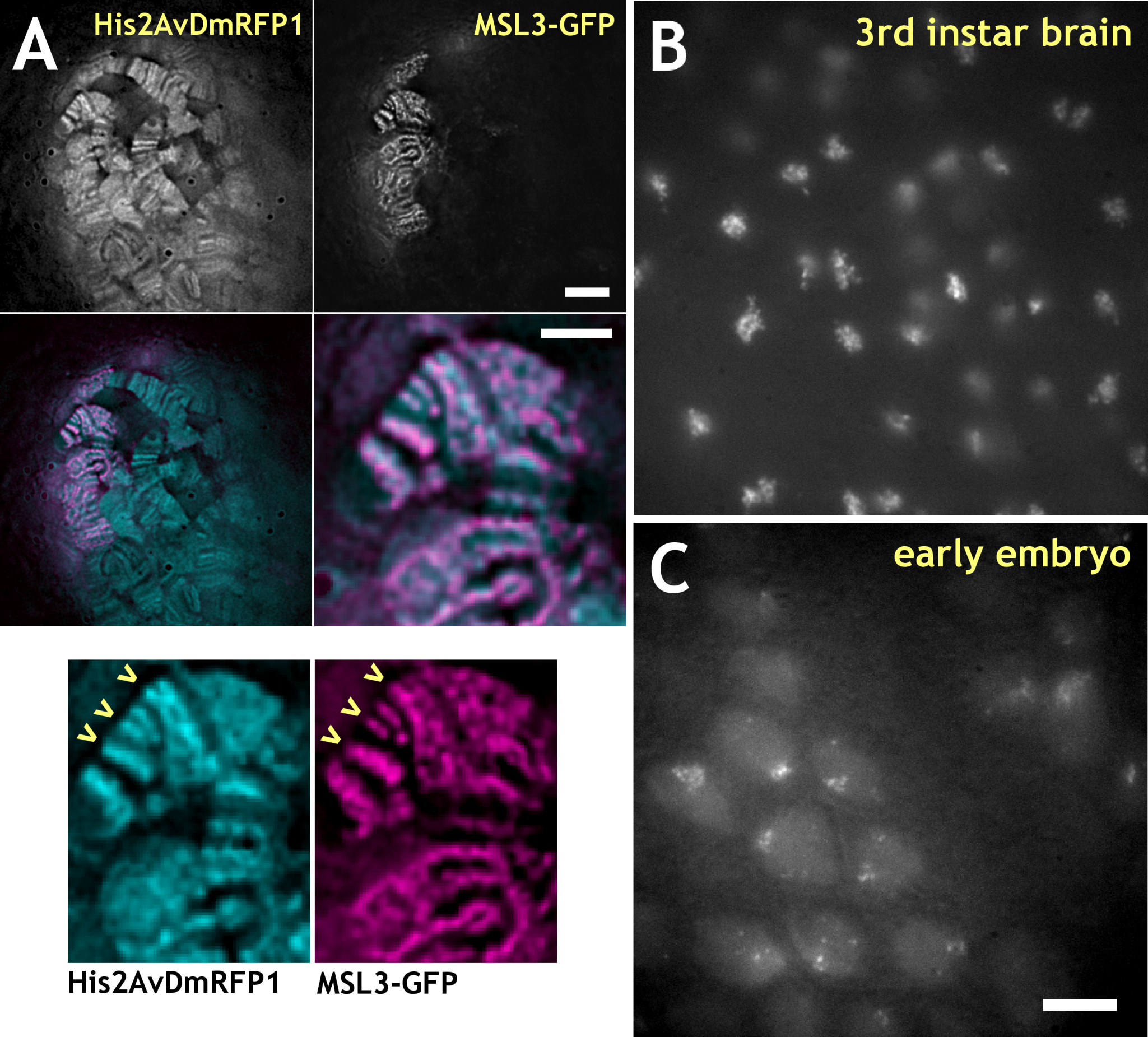

Supplement: Figure S1 — Examples of polytene chromosomes, embryos, and tissues from a transgenic fly line carrying MSL3-GFP and His2AvDmRFP1. (A) Live polytene nuclei isolated from 3rd instar larvae of yw; [w+ M3-GFP]; [w+ M3-GFP] line. Top row: His2AvDmRFP1 and MSL3-GFP channels, respectively; middle row: superimposed, pseudo-colored images, with a close-up of 3-fold higher magnification; bottom row: His2AvDmRFP1 and MSL3-GFP are largely, though not perfectly, co-localized. The arrowheads show two co-localized bands in both channels. (B) Live brain of 3rd instar larvae expressing MSL3-GFP. GFP signal of many X chromosomes shows radially non-uniform organization with reduced intensity in the middle of the signal. (C) Live embryos during gastrulation, cell cycle 15 with compact MSL3-GFP signals. Bars: 5 µm – (A) (top row), (C); 2 µm – (A) (middle row). (3.39 MB TIF) [file pbio.1000574.s001.tif]

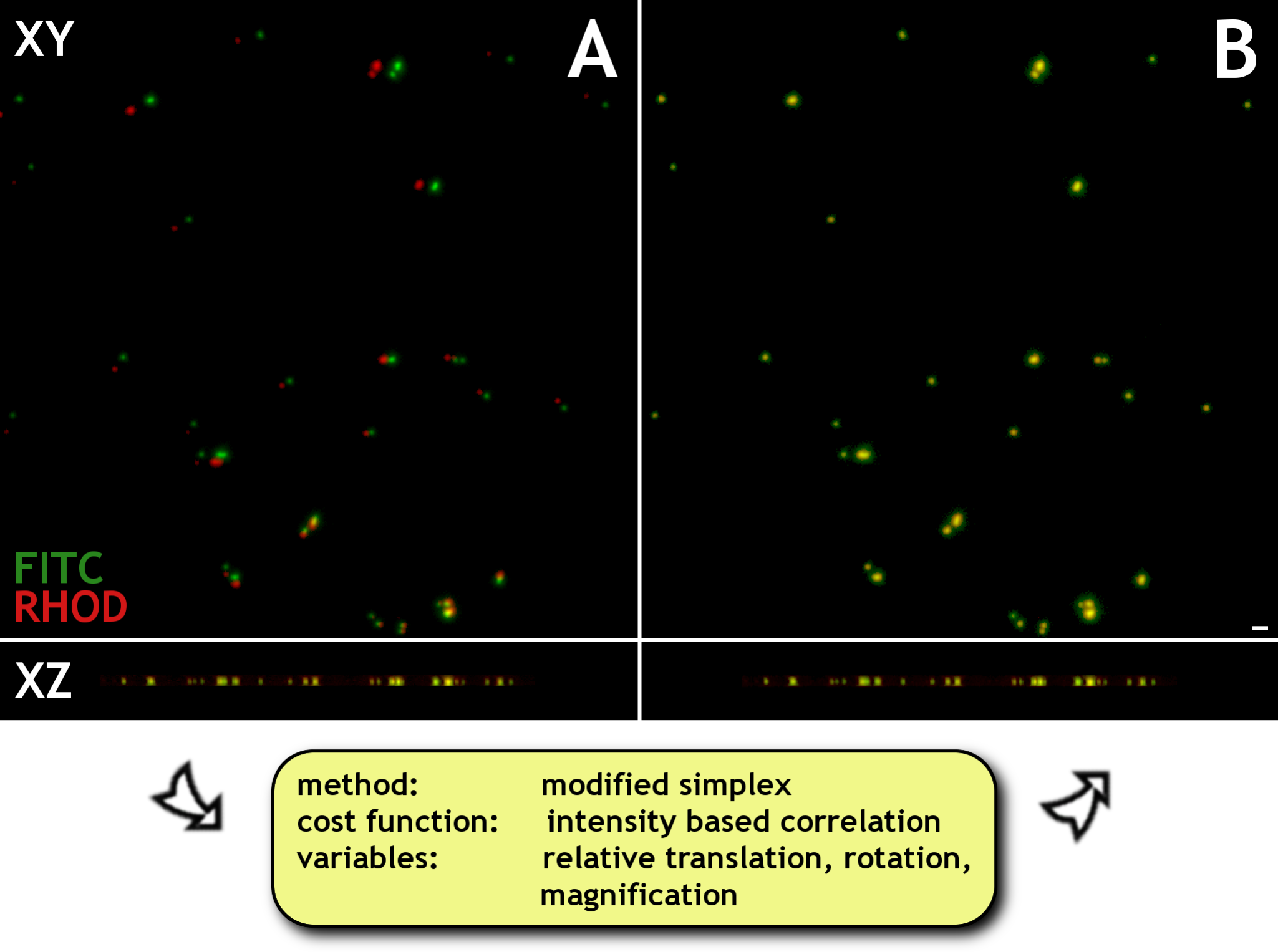

Supplement: Figure S2 — Digital, postimaging alignment of different color channels is necessary to exclude the contributions of chromatic aberrations, relative differences in CCD camera adjustments, such as translations, rotations and magnification, and variations in the optical paths of the color channels. Shown are the FITC and RHOD channels before (A) and after (B) alignment: the RHOD channel was translated, rotated, and magnification compensated to match the FITC channel. Top panels show the XY projections of a 3D bead data set, bottom panels – XZ projections. Bar: 1 µm. (0.31 MB TIF) [file pbio.1000574.s002.tif]

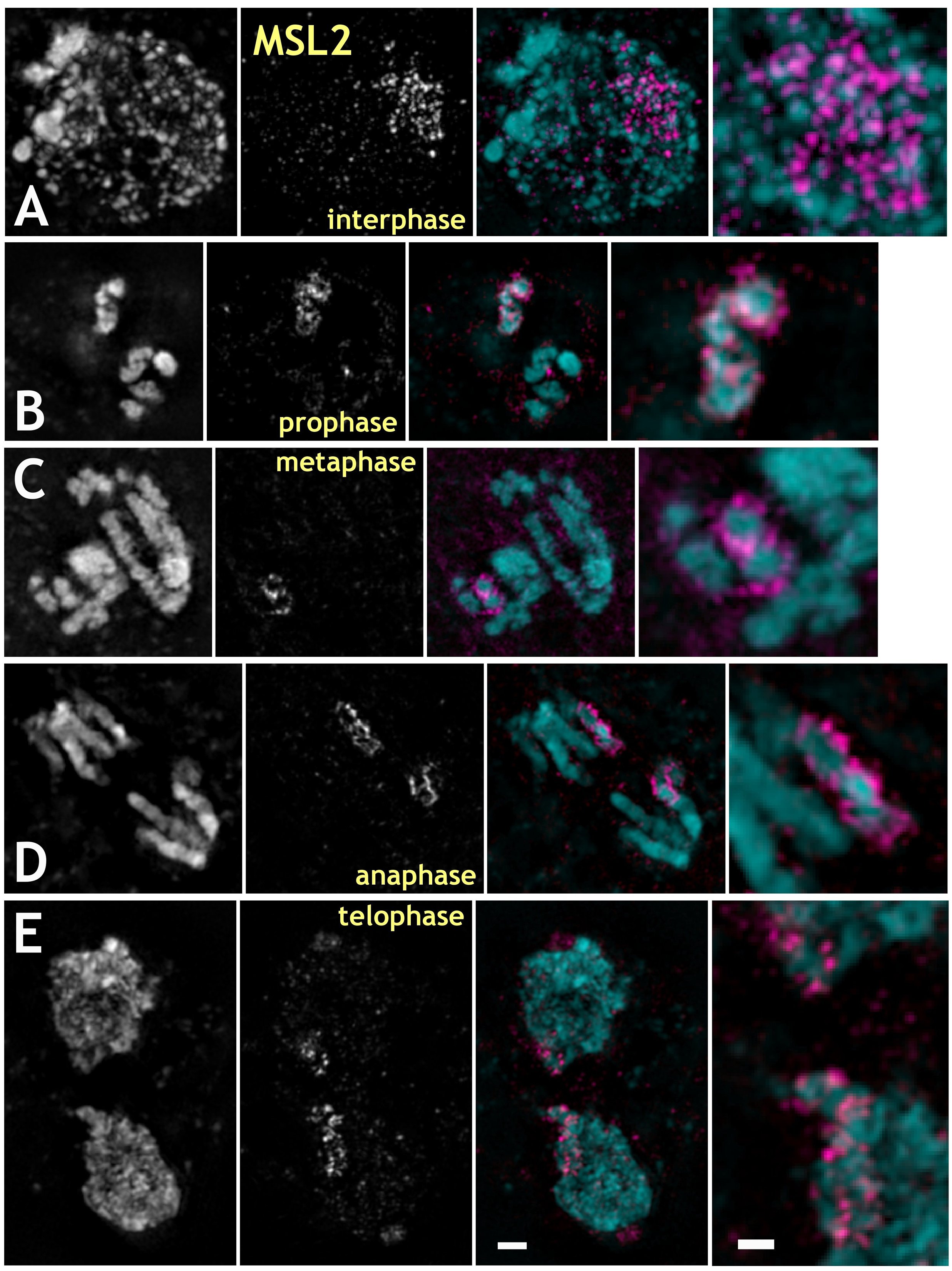

Supplement: Figure S3 — Actively transcribed sequences target to the periphery of chromosomes at different stages of mitosis and at interphase in fixed, anti-MSL2 antibody stained cells of embryonic cultures isolated from Oregon R line and imaged with SIM. Despite overlap between anti-MSL2 and DAPI signals, some MSL2 stayed outside the DAPI-labeled chromatin. For each row, (A) through (E), shown are from left to right DAPI, anti-MSL2, pseudo-colored DAPI (cyan) and anti-MSL2 (magenta) superimposed, and a 2.5-fold increased magnification of the antibody labeled chromosome arm. (A) interphase; (B) prometaphase; (C) metaphase; (D) in anaphase, the anti-MSL2 signal was 400–600 nm in diameter with the DAPI-stained chromatid diameter of 400–500 nm. (E) telophase. Bars: 1 µm – whole cell images; 0.5 µm – expanded regions. (7.13 MB TIF) [file pbio.1000574.s003.tif]

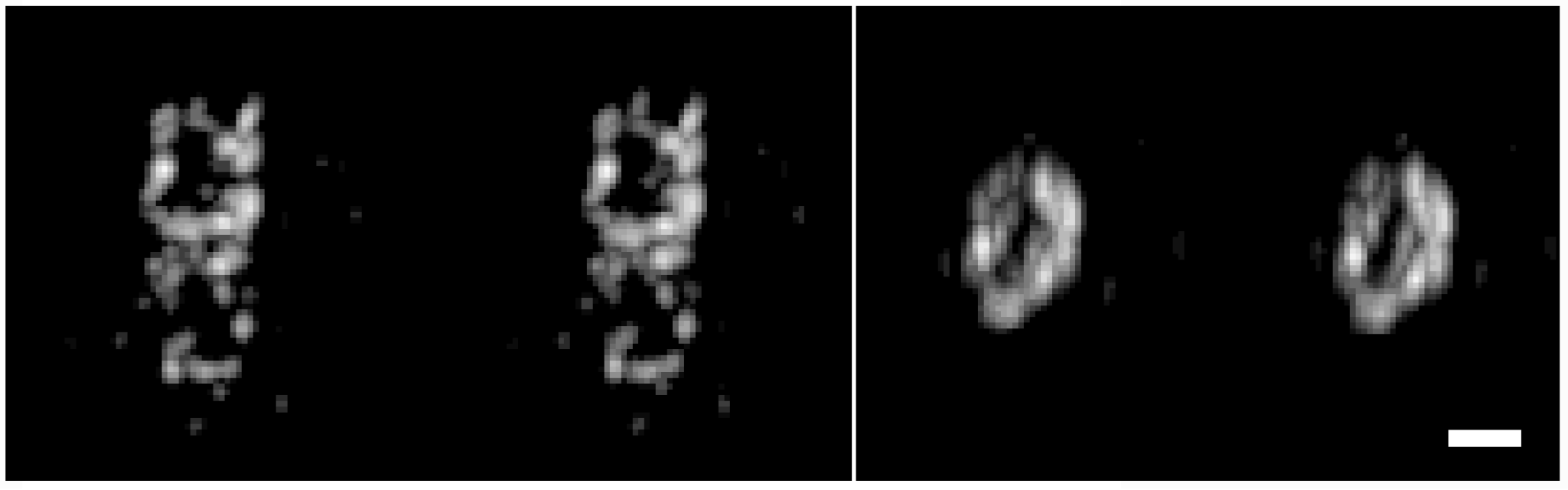

Supplement: Figure S4 — Stereo-pairs of anti-GFP stained, SIM-imaged (single sister chromatid) chromosomes in fixed cells isolated from MSL3-GFP expressing embryos. Only the euchromatic arm of X chromosome is labeled: side view with telomeres at the bottom (left) and axial view with a staining-free channel within an anaphase chromatid (right). Bar: 0.5 µm. (0.11 MB TIF) [file pbio.1000574.s004.tif]

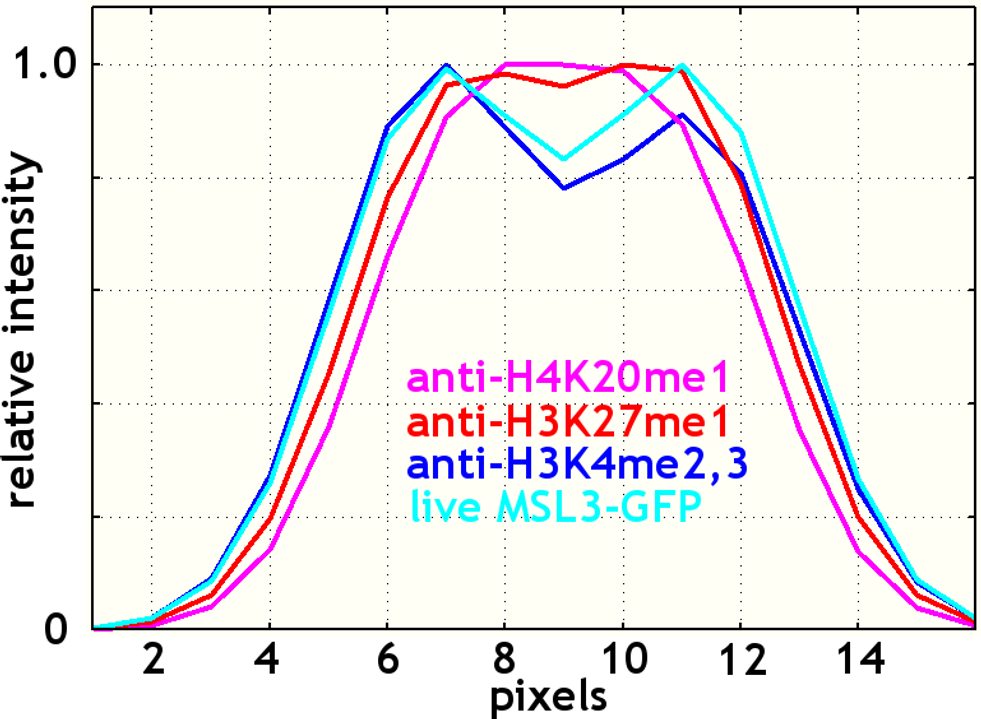

Supplement: Figure S5 — Immunofluorescence staining against different histone modifications and the MSL3-GFP signal have different widths and intensity distributions relative to chromosomal DNA. The intensities of individual profiles in each group was normalized, then averaged and plotted to demonstrate differences both in the relative widths and signal distributions. Each individual profile was an average over a straight linear segment of a chromosomal arm 15 pixels or about 1200 nm long. Anti-H3K4me2,3 and live MSL3-GFP signals had equal widths, ∼630 nm (std 91 nm), pronounced depletion of the signal at the core, and well-separated and coinciding peaks of peripheral signals. Anti-H3K27me1 was narrower than the first two, 533 nm (std 108) and had barely resolved peripheral signals with almost no drop of the intensity at the core. Anti-H4K20me1 signal was 500 nm (std 67) wide and had no dip at the core, similar in the profile to DAPI staining and suggesting that it stained more internal regions of chromosomes compared to MSL3-GFP or the other antibody signals. Normalization of individual profiles by the chromosome width measured with DAPI or His2AvDmRFP1 signals produced similar averaged values. (0.22 MB TIF) [file pbio.1000574.s005.tif]

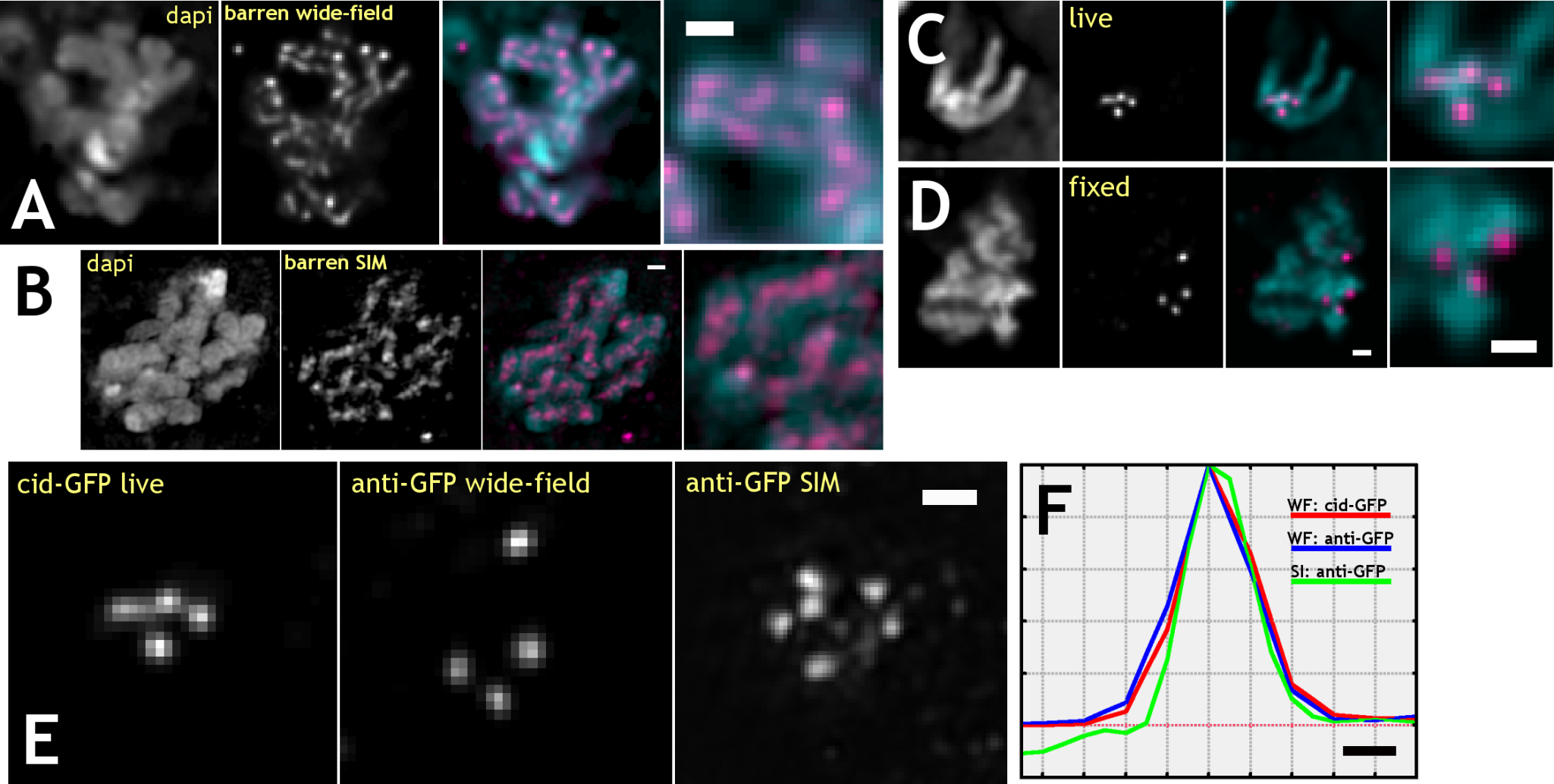

Supplement: Figure S6 — Mitotic chromatin is not refractory to immunofluorescence. Wide-field imaged metaphase (A) and SIM-imaged anaphase (B) chromosomes stained with anti-barren antibodies. From left to right: DAPI, anti-barren antibody, pseudo-colored and superimposed DAPI (cyan) and anti-barren (magenta), 2.5-fold higher magnification of the superimposition. The dimensions and the shapes of the centromeres are comparable in live and fixed cells. (C) Live cells, from left to right: His2AvDmRFP1, cid-GFP, cid-GFP (magenta), and His2AvDmRFP1 (cyan) combined. (D) Fixed cells, from left to right: DAPI, anti-GFP antibody, anti-GFP antibody (magenta), and DAPI (cyan) combined. (E) The appearance and dimensions of centromeres do not depend on labeling and imaging methods. From left to right: cid-GFP imaged with wide-filed microscopy, anti-GFP antibody staining imaged with wide-filed microscopy (both expanded from panels C and D), and centromeres after anti-GFP antibody staining of a fixed cell imaged with SIM. All non-SIM images were deconvolved. (F) Intensity line profiles across centromere images with different modalities are comparable at FWHM. Bars: 0.5 µm – (A), (B), (D) (centromere images); 0.1 µm – line profiles in (F). (0.94 MB TIF) [file pbio.1000574.s006.tif]
