# Supplementary material for: Evidence of Activity-Specific, Radial Organization of Mitotic Chromosomes in Drosophila
Source: PLoS Biol. 2011 Jan 11;9(1):e1000574. doi: 10.1371/journal.pbio.1000574 (PMC3019107; doi:10.1371/journal.pbio.1000574)
Supplement: Table S1 — The results of hypothesis testing with the null hypothesis of pair-wise equal means and different alternative hypotheses are in agreement with the observed variations in the distribution of histone modifications across the chromosome width. One-sided alternative hypothesis H1 was that the mean of the first data set was greater than the mean of the second data set in the same row. Two-sided alternative hypothesis H1 was that the mean of the first data set was less or greater than the mean of the second data set in the same row. The results of hypothesis testing support rejection of the null hypothesis of equal means for the following pairs: H3K4me2,3 and H4K20me1; H3K4me2,3 and H3K27me1; H4K20me1 and MSL3-GFP; MSL3-GFP and H3K27me1. Rejection of the null hypothesis of equal means is not supported for the following pairs: H3K4me2,3 and MSL3-GFP; H3K27me1 and H4K20me1. This is in agreement with our conclusions based on visual observations and examination of individual profiles. (0.11 MB DOC) [file pbio.1000574.s007.doc]

**Table S1. The results of hypothesis testing with the null hypothesis of pair-wise equal means and different alternative hypotheses are in agreement with the observed variations in the distribution of histone modifications across the chromosome width.**

| Data sets for the null hypothesis of equal means | p-value  one-sided H_1_ | p-value  two-sided H_1_ |
| --- | --- | --- |
| H3K4me2,3 and H4K20me1 | 1.59e-08 | 3.18e-08 |
| H3K4me2,3 and H3K27me1 | 9.18e-05 | 1.84e-04 |
| H3K4me2,3 and MSL3-GFP | 3.11e-01 | 6.22e-01 |
| H3K27me1 and H4K20me1 | 2.17e-02 | 4.34e-02 |
| H4K20me1 and MSL3-GFP | 9.35e-06 | 1.87e-05 |
| MSL3-GFP and H3K27me1 | 1.54e-03 | 3.07e-03 |

One-sided alternative hypothesis H_1_ was that the mean of the first data set was greater than the mean of the second data set in the same row. Two-sided alternative hypothesis H_1_ was that the mean of the first data set was less or greater than the mean of the second data set in the same row.

The results of hypothesis testing support rejection of the null hypothesis of equal means for the following pairs: H3K4me2,3 and H4K20me1; H3K4me2,3 and H3K27me1; H4K20me1 and MSL3-GFP; MSL3-GFP and H3K27me1. Rejection of the null hypothesis of equal means is not supported for the following pairs: H3K4me2,3 and MSL3-GFP; H3K27me1 and H4K20me1. This is in agreement with our conclusions based on visual observations and examination of individual profiles.
