# Supplementary material for: Evidence of Activity-Specific, Radial Organization of Mitotic Chromosomes in Drosophila
Source: PLoS Biol. 2011 Jan 11;9(1):e1000574. doi: 10.1371/journal.pbio.1000574 (PMC3019107; doi:10.1371/journal.pbio.1000574)
Supplement: Text S1 — Supporting text contains extra details and discussion of methods and supporting figures and videos. Supporting information includes detailed protocols of preparation of Drosophila embryonic cultures and dissected tissues, fixation and immunostaining procedure, and fluorescence microscopy imaging and image processing. (0.14 MB DOC) [file pbio.1000574.s008.doc]

**Supporting Text**

In an attempt to fill the gap between the biochemical studies of mitotic chromatin with studies on its larger-scale structure we decided to combine structural and functional studies by selectively marking a subset of sequences of otherwise uniformly packed, featureless bulk chromatin inside cells. Previous studies of the chromosome structure at specific chromatin loci used either artificially introduced DNA sequences or concentrate on individual small regions limiting the general view. Artificially introduced sequences are known to locally modify the properties of chromatin, thus limiting the possibility of interpretation and generalization of the contribution of native chromatin (Y.Strukov, unpublished results). The transgenic chromatin organization may vary depending on the integration site. Small, individual regions, on the other hand, might not provide complete description of chromatin structure due to significant contribution of stochastic mechanisms into mitotic chromatin structure, requiring large, technically challenging number of loci for generalization. In addition, non-amplified, relatively short single inserts (several tens of kbp) are too small to image within large-scale chromatin features (e.g., a mitotic chromosome band of several Mbp). To avoid problems connected with transgenic regions, we studied the organization and distribution of native chromatin loci.

As a source of chromosomes, Drosophila embryonic and larval tissues also meet several criteria important for structural studies: (a) diploid cells have 3 pairs of large chromosomes and a pair of tiny 4^th^ chromosomes which allows unobstructed imaging and analysis of individual chromosomes in mitosis; (b) cells have a relatively short cell cycle, ~50 min, thus eliminating extended periods of imaging under detrimental conditions and facilitating observations of cell cycle-related chromatin dynamics for live imaging; and, importantly; (c) specific, non-disturbing labeling of native regions of chromatin that, on the one hand, are representative for generalization of the observed structure, and, on the other hand, are small compared to the total DNA for easy isolation and tracking.

**Supporting Methods**

***Embryos Cultures and Tissues***

Embryos collected for 1 hour on an agar plate were aged for 4 hours at room temperature. After a rinse with water, treatment with 30% household bleach for 1 min and a rinse with water for 2 min, embryos were Dounce homogenized in Chan and Gehring’s Balances Saline (3.2 g/l NaCl, 3.0 g/l CaCl_2_ **.** 2H_2_O, 0.69 g/l MgSO_4_ **.** 7H_2_O, 1.79 g/l Tricine buffer (pH 7.0), 3.6 g/l glucose, 17.1 g/l sucrose, 1.0 g/l BSA (Fraction V) supplied with 2% defined FBS (HyClone Cat No: SH30070.02). After a series of centrifugations with clinical centrifuge at +4^o^C and washes, the pellet was resuspended in 0.5 ml of the medium and spread over poly-L-lysine treated cover slips. For poly-L-lysine treatment, acid-washed cover slips were incubated in 1 mg/ml poly-L-lysine (Sigma-Aldrich, Cat. No: P2636) solution buffered at pH 11 for 1 hr at RT, washed with distilled water and air dried for 1 hr at RT before applying freshly made embryonic cultures. Isolated primary cultures had to be deposited on poly-L-lysine coated cover slips to minimize the drift of cells during live or SIM imaging and to avoid cell loss during immunofluorescence washes. Embryonic culture suspensions on cover slips were incubated for 30 min in a wet chamber before fixation. Brains for live observations were dissected from 3^rd^ instar larvae in D-22 medium (Sigma-Aldrich, St. Louis, MO) supplied with 5% heat inactivated FBS (HyClone), placed with optical lobes down on an acid-washed cover slip in a drop of the medium and covered with a gas-permeable membrane. Fresh embryonic cultures provided sufficient numbers of mitotic cells for immunofluorescence, therefore mitotic blockers were not used to avoid artifacts in the chromosome structure due to over-condensation [1].

***Immunostaining***

A mixture of primary antibodies from rabbit and mouse and a mixture fluorophore-labeled secondary antibodies against rabbit and mouse were used for double-antibody labeling experiments. A monolayer of embryonic culture was permeabilized for 30 sec at RT in buffer C (0.25 M sucrose, 10 mM PIPES pH 7.0, 1.5 mM MgCl_2_, 1 mM CaCl_2_) supplemented with 0.1% Triton X-100, fixed with 1.6% freshly made formaldehyde in buffer C supplemented with 0.02% Tween-20 for 10 min at RT, rinsed 3 times in buffer C with 0.02% Tween-20, washed 3 times with buffer A* (80 mM KCl, 20 mM NaCl, 2 mM EDTA, 0.5 mM EGTA, 0.5 mM spermidine, 0.2 mM spermine adjusted to pH 7.0 and supplemented with 0.02% Tween-20), then incubated 3 times for 5 min with 20 mM glycine solution in buffer A*. After a 5 min wash with buffer A*, cover slips were incubated for 1 hour at +4^o^C in 5% NGS (Normal Goat Serum) in buffer A* and then washed with buffer A* for 5 min at RT. Primary antibodies were applied in 1/200 to 1/10000 dilutions in buffer A (buffer A* without Tween-20) supplemented with 0.1% Triton X-100 and 2% Normal Goat Serum overnight at +4^o^C. Secondary antibodies were applied in the same buffer at 1/2000 dilution for 3 hours at +4^o^C after three 10-min washes with buffer A containing 0.1% Triton X-100. After three 5-min washes with 0.1% Triton X-100 in buffer A, cover slips were stained with 5 ng/ml DAPI in buffer A* and mounted in VectaShield medium (H-1000, Vector Laboratories, Burlingame, CA). In a different line of experiments immunostaining was done with PBS* (Ca- and Mg-free PBS with 5 mM MgCl_2_, and 0.1 mM EDTA) instead of buffers A and C with the same results. Substitution of buffer C with buffer A produced the same results.

***Imaging and Image Processing***

100x NA1.4 oil objective lens (Olympus) was used for imaging of fixed samples and live cultures and tissues. 403 nm, 488 nm, and 532 nm lasers were used for excitation. Fluorescence was recorded with three emission channels: DAPI (460±25nm), FITC (515±15nm), RHOD (590±15nm). Each emission channel had a dedicated CCD camera. All recorded images were 512x512 in size. For wide-field deconvolution multi-color experiments, the color channels were recorded sequentially onto different CCD cameras. DAPI emission was recorded onto the FITC camera, GFP and green fluorescence-labeled antibody samples were recorded onto the FITC camera, and RFP and red fluorescence-labeled antibody samples were recorded onto the RHOD camera. For SIM experiments, the DAPI signal was recorded onto the DAPI camera. Only individual, well-separated sister chromatids could be used for scoring and downstream analysis with automated scripts. Therefore, all chromosomes on each slide that met the following criteria were assayed: first, they showed clear separation of sister chromatids within mitotic cells and, second, they were not obscured by other chromosomes inside the same mitotic cell. For measurements of chromosome widths and label distribution, straight, linear segments of 15 pixels, or ~1.2 μm long, were found, one per chromosome. The background was subtracted during deconvolution. The intensities of DAPI, His2AvDmRFP1, MSL3-GFP, or antibody stainings were summed along the chromosomal axes by projection using procedures in ImageJ. The widths of the resulting profiles perpendicular to the axes were then measured as full width at half maximum and averaged for each group of chromosomes from different labeling methods. Some profiles contained two lobes coming from preferentially peripheral localization of the specific signal with an intensity drop at the cores. To measure the separation of the peaks of peripheral signals, a sum of two Gaussian functions of fixed width was fit to each profile with an optimization procedure implemented in GNU Octave. The positions and heights of each Gaussian peak were optimization parameters; the widths of Gaussian functions were assumed to be 250 nm, which is a diameter of a diffraction-limited, PSF-blurred point source. The summary of the measurements of chromosomes from different experiments is shown in Table 1. Formal hypothesis testing was undertaken to support rejection of the null hypothesis of equal means for each pair of data sets. The results are summarized in Table S1. The results of hypothesis testing support rejection of the null hypothesis of equal means for the following pairs: H3K4me2,3 and H4K20me; H3K4me2,3 and H3K27me1; H4K20me1 and MSL3-GFP; MSL3-GFP and H3K27me1. Rejection of the null hypothesis of equal means is not supported for the following pairs: H3K4me2,3 and MSL3-GFP; H3K27me1 and H4K20me1. This is in agreement with our conclusions based on visual observations and examination of individual profiles. Cohen's effect size varied from 1.2 to 2.4 for different pairs of our data sets, which meant that 10-15 chromosomes per data set was sufficient to support rejection of the null hypothesis of equal means with the significance level of at most 0.01, the test power of 0.8 for one- or two-sided alternative hypotheses. For testing the null hypothesis of equal means we used Welch's t-test, which is intended for two data sets from normal distributions of unknown means and unknown and not necessary equal variances. We assumed that samples within the data sets were normally distributed. The mean of the widths of chromosomes and their variance were similar in different data sets. The null hypothesis of equal widths of chromosomes of different data sets measured after labeling with DAPI or His2AvDmRFP1 cannot be rejected.

***Correction of chromatic aberrations and color channel misalignment***

For correction of chromatic aberration of the objective lens and variations in CCD camera specifications, Z-stacks of 100 nm multi-wavelength fluorescent beads were recorded with the same setup as the chromosome samples and used for calculation of alignment parameters, separately for wide-field and SIM due to differences in optics setups. Differences in translation, rotation, anisotropic and isotropic magnifications were calculated through optimization (Fig. S2). The bead data were filtered with laplacian of Gaussian (“mexican hat”) for increased contrast. The cost function for Nelder-Mead modified simplex method [2] was intensity-based cross-correlation calculated after background subtraction and intensity normalization; five restarts with newly generated starting simplex were used for each alignment, and the best fittings were selected. The maximum number of simplex iterations was 100; simplex iterations were stopped when consecutive cost function changes were less then 10^-6^. Calculated relative translations of channels in X and Y were 10-100 pixels, translations in Z were below 2 pixels, rotations between different channels were on the order of 1 degree, and magnification differences were less than 0.3%. Actual magnification differences were calculated in the optimization procedure. Magnification differences between color channels were compensated for despite their minute scale. Application of calculated alignment parameters to multi-color bead Z-stacks gave superimposition of beads uniform over the overlapping regions. Beads close to the center of a 512 pixels by 512 pixels size image and beads located at the periphery of the image were co-localized indicating elimination of magnification differences. Custom Python scripts were used for calculation and application of calculated alignment parameters with the sub-pixel accuracy.

**Supporting Figures**

Fig. S1 Examples of polytene chromosomes, embryos and tissues from a transgenic fly line carrying MSL3-GFP and His2AvDmRFP1. (A) Live polytene nuclei isolated from 3^rd^ instar larvae of yw; [w+ M3-GFP]; [w+ M3-GFP] line. Top row: His2AvDmRFP1 and MSL3-GFP channels, respectively; middle row: superimposed, pseudo-colored images, with a close-up of 3-fold higher magnification; bottom row: His2AvDmRFP1 and MSL3-GFP are largely, though not perfectly, co-localized. The arrowheads show to co-localized bands in both channels. (B) Live brain of 3^rd^ instar larvae expressing MSL3-GFP. GFP signal of many X chromosomes shows radially non-uniform organization with reduced intensity in the middle of the signal. (C) Live embryos during gastrulation, cell cycle 15 with compact MSL3-GFP signals. Bars: 5 μm – A (top row), C; 2 μm – A (middle row).

Fig S2 Digital, postimaging alignment of different color channels is necessary to exclude the contributions of chromatic aberrations, relative differences in CCD camera adjustments, such as translations, rotations and magnification, and variations in the optical paths of the color channels. Shown are the FITC and RHOD channels before (A) and after (B) alignment: the RHOD channel was translated, rotated and magnification compensated to match the FITC channel. Top panels show the XY projections of a 3D bead data set, bottom panels – XZ projections. Bar: 1 μm

Fig. S3 Actively transcribed sequences target to the periphery of chromosomes at different stages of mitosis and at interphase in fixed, anti-MSL2 antibody stained cells of embryonic cultures isolated from Oregon R line and imaged with SIM. Despite overlap between anti-MSL2 and DAPI signals, some MSL2 stayed outside the DAPI-labeled chromatin. For each row, A through E, shown are from left to right DAPI, anti-MSL2, pseudo-colored DAPI (cyan) and anti-MSL2 (magenta) superimposed, and a 2.5-fold increased magnification of the antibody labeled chromosome arm. (A) interphase; (B) prometaphase; (C) metaphase; (D) in anaphase, the anti-MSL2 signal was 400-600 nm in diameter with the DAPI-stained chromatid diameter of 400-500 nm. (E) telophase. Bars: 1 μm – whole cell images, 0.5 μm – expanded regions.

Fig. S4 Stereo-pairs of anti-GFP stained, SIM-imaged (single sister chromatid) chromosomes in fixed cells isolated from MSL3-GFP expressing embryos. Only the euchromatic arm of X chromosome is labeled: side view with telomeres at the bottom (left) and axial view with a staining-free channel within an anaphase chromatid (right). Bar: 0.5 μm

Fig. S5 Immunofluorescence staining against different histone modifications and the MSL3-GFP signal have different widths and intensity distributions relative to chromosomal DNA. The intensities of individual profiles in each group was normalized, then averaged and plotted to demonstrate differences both in the relative widths and signal distributions. Each individual profile was an average over a straight linear segment of a chromosomal arm 15 pixels or about 1200 nm long. Anti-H3K4me2,3 and live MSL3-GFP signals had equal widths, ~630 nm (std 91 nm), pronounced depletion of the signal at the core, and well-separated and coinciding peaks of peripheral signals. Anti-H3K27me1 was narrower than the first two, 533 nm (std 108), had barely resolved peripheral signals with almost no drop of the intensity at the core. Anti-H4K20me1 signal was 500 nm (std 67) wide and had no dip at the core similar in the profile to DAPI staining and suggesting that it stained more internal regions of chromosomes compared to MSL3-GFP or the other antibody signals. Normalization of individual profiles by the chromosome width measured with DAPI or His2AvDmRFP1 signals produced similar averaged values.

Fig. S6 Mitotic chromatin is not refractory to immunofluorescence. Wide-field imaged metaphase (A) and SIM-imaged anaphase (B) chromosomes stained with anti-barren antibodies. From left to right: DAPI, anti-barren antibody, pseudo-colored and superimposed DAPI (cyan) and anti-barren (magenta), 2.5-fold higher magnification of the superimposition. The dimensions and the shapes of the centromeres are comparable in live and fixed cells. (C) Live cells - from left to right: His2AvDmRFP1, cid-GFP, cid-GFP (magenta) and His2AvDmRFP1 (cyan) combined. (D) Fixed cells - from left to right: DAPI, anti-GFP antibody, anti-GFP antibody (magenta) and DAPI (cyan) combined. (E) The appearance and dimensions of centromeres do not depend on labeling and imaging methods. From left to right: cid-GFP imaged with wide-filed microscopy, anti-GFP antibody staining imaged with wide-filed microscopy (both expanded from panels C and D), centromeres after anti-GFP antibody staining of a fixed cell imaged with SIM. All non-SIM images were deconvolved. (F) Intensity line profiles across centromeres images with different modalities are comparable at FWHM. Bars: 0.5 μm – A, B, D (centromere images); 0.1 μm – line profiles in F.

**Supporting Movies**

Movie S1 Mitotic condensation of chromatin in embryonic cultures isolated from msl3GFP, His2AvDmRFP1; msl3GFP line. Maximum intensity projections of 6 optical sections 0.5 μm apart; images were taken once every 40 sec.

Movie S2 A Z-stack of fixed metaphase cell in embryonic culture isolated from msl3GFP, His2AvDmRFP1; msl3GFP line: optical sections are 150 nm apart.

Movie S3 3D reconstruction of anti-GFP staining of fixed anaphase X chromosome of MSL3-GFP line imaged with SI.

Movie S4 Live decondensation of MSL3-GFP labeled chromosome in larval brains from mid-anaphase through interphase: 178 time points, 10 sec apart.

1. Maeshima, K. and U.K. Laemmli, *A two-step scaffolding model for mitotic chromosome assembly.* Dev Cell, 2003. **4**(4): p. 467-80.

2. Nelder, J. A., Mead, R. *A simplex method for function minimization.* Computer

Journal 7(1965) 308-313.
